# ALS mutant FUS proteins are recruited into stress granules in induced pluripotent stem cell-derived motoneurons

**DOI:** 10.1242/dmm.020099

**Published:** 2015-07-01

**Authors:** Jessica Lenzi, Riccardo De Santis, Valeria de Turris, Mariangela Morlando, Pietro Laneve, Andrea Calvo, Virginia Caliendo, Adriano Chiò, Alessandro Rosa, Irene Bozzoni

**Affiliations:** 1 Center for Life Nano Science, Istituto Italiano di Tecnologia, Viale Regina Elena 291, Rome 00161, Italy; 2 Department of Biology and Biotechnology Charles Darwin, Sapienza University of Rome, P.le A. Moro 5, Rome 00185, Italy; 3 Amyotrophic Lateral Sclerosis Center, Rita Levi Montalcini Department of Neuroscience, University of Turin, 10126 Turin, Italy; 4 Dermatologia Chirurgica AOU Città della Salute e della Scienza, 10126 Turin, Italy; 5 Institute Pasteur Fondazione Cenci-Bolognetti, Sapienza University of Rome, P.le A. Moro 5, Rome 00185, Italy

**Keywords:** ALS, FUS, TALE nucleases, iPSCs

## Abstract

Patient-derived induced pluripotent stem cells (iPSCs) provide an opportunity to study human diseases mainly in those cases for which no suitable model systems are available. Here, we have taken advantage of *in vitro* iPSCs derived from patients affected by amyotrophic lateral sclerosis (ALS) and carrying mutations in the RNA-binding protein FUS to study the cellular behavior of the mutant proteins in the appropriate genetic background. Moreover, the ability to differentiate iPSCs into spinal cord neural cells provides an *in vitro* model mimicking the physiological conditions. iPSCs were derived from FUS^R514S^ and FUS^R521C^ patient fibroblasts, whereas in the case of the severe FUS^P525L^ mutation, in which fibroblasts were not available, a heterozygous and a homozygous iPSC line were raised by TALEN-directed mutagenesis. We show that aberrant localization and recruitment of FUS into stress granules (SGs) is a prerogative of the FUS mutant proteins and occurs only upon induction of stress in both undifferentiated iPSCs and spinal cord neural cells. Moreover, we show that the incorporation into SGs is proportional to the amount of cytoplasmic FUS, strongly correlating with the cytoplasmic delocalization phenotype of the different mutants. Therefore, the available iPSCs represent a very powerful system for understanding the correlation between FUS mutations, the molecular mechanisms of SG formation and ALS ethiopathogenesis.

## Abstract (precis)

**Summary:** Mutated FUS protein is aberrantly delocalized and recruited into stress granules in iPSC-derived motoneurons, which provide a new model system for amyotrophic lateral sclerosis.

## INTRODUCTION

Amyotrophic lateral sclerosis (ALS) is a fatal neurodegenerative disease caused by loss of motoneurons (MNs) in the spinal cord and brain, leading to progressive muscle atrophy. About 10% of ALS cases are familial (fALS), whereas the rest is sporadic (sALS). Several genes have been linked to familial and sporadic ALS. Among them, Cu/Zn superoxide dismutase 1 (*SOD1*), chromosome 9 open reading frame 72 (*C9orf72* – Human Gene Nomenclature Database), Tar DNA-binding protein 43 (*TDP-43*; *TARDBP* – Human Gene Nomenclature Database) and fused in sarcoma/translocated in liposarcoma (*FUS/TLS* or *FUS*) account for most fALS cases (Renton et al., 2014). FUS and TDP-43 are both RNA-binding proteins with a role in multiple steps of RNA processing (Lagier-Tourenne et al., 2010). This suggests a common mechanism underlying their involvement in ALS. However, a clear correlation between the genetic defect and the physiopathology of the disease remains elusive. Both FUS and TDP-43 are mainly localized in the nucleus but shuttle between the nucleus and the cytosol (Zinszner et al., 1997; Ayala et al., 2008). A hallmark of the pathology is the presence of cytoplasmic inclusions of mutated proteins in the brain (including frontal cortex, substantia nigra, amygdala and cingulate gyrus) and spinal cord of FUS (Kwiatkowski et al., 2009; Vance et al., 2009) and TDP-43 (Yokoseki et al., 2008; Van Deerlin et al., 2008) patients. Many ALS-associated FUS mutations disrupt the function of the C-terminal PY domain, which serves as a nuclear localization signal (NLS) (Vance et al., 2009, 2013; Gal et al., 2011; Dormann et al., 2010; Bosco et al., 2010; Ito et al., 2010; Kino et al., 2011). Defects in nuclear import, leading to aberrant cytoplasmic localization of FUS, have been proposed as the initial step in ALS pathogenesis (Bentmann et al., 2013).

So far, it is unclear whether reduced MN survival in ALS patients is due to loss of a nuclear function or gain of a still unidentified toxic function in the cytoplasm, or a combination of both (Lagier-Tourenne et al., 2010). Increased oxidative stress is thought to play a role in ALS pathogenesis, and FUS and TDP-43 cytoplasmic inclusions co-localize with stress granule (SG) markers in ALS patients (Ferrante et al., 1997; Liu-Yesucevitz et al., 2010; Bentmann et al., 2012). SGs form in the cytoplasm upon exposure to several kinds of stress. They represent mRNA storage and sorting compartments that protect the cell by allowing prioritized translation of stress-response genes. FUS, TDP-43 and other RNA-binding proteins involved in ALS (such as EWS/EWSR1, TAF15 and ATXN2) can be found in SGs and/or regulate SG assembly (Bentmann et al., 2013). It has been proposed that SGs play a role in neurodegeneration as possible precursors of pathological inclusions (Bentmann et al., 2013; Wolozin, 2012). Even though transient SGs have a protective function in normal neurons, mutations in their components might convert them into overly stable structures (Wolozin, 2012). In the case of ALS patients with TDP-43 and FUS mutations, trapping of these pleiotropic RNA-binding proteins in permanent inclusions might result in loss of crucial regulation of RNA splicing, maturation and transport, and/or gain of a toxic function.

So far, the molecular analysis of ALS pathogenesis has been hampered by the lack of suitable cell model systems. Reprogramming of human somatic cells into induced pluripotent stem cells (iPSCs) provides a unique opportunity for disease modeling *in vitro*. iPSCs can be derived from patients harboring a disease-associated mutation (patient-specific iPSCs, PS-iPSCs) and then differentiated into (virtually) any cell type (Stadtfeld and Hochedlinger, 2010). Thus, iPSC-based models represent a new powerful tool to study the mechanisms underlying the pathophysiology of neurodegenerative diseases in the context of human neurons (Sandoe and Eggan, 2013). The potential of iPSCs can be further expanded by the possibility to edit their genome by site-directed mutagenesis. Several tools are now available for genetic engineering in iPSCs, including systems based on transcription activator-like effector nucleases (TALENs) (Hockemeyer et al., 2011) and on clustered regularly interspaced short palindromic repeats (CRISPR)/Cas9 (Mali et al., 2013). Several groups have reported the characterization of iPSCs derived from fALS individuals with mutations in the *SOD1* and *TDP-43* genes (Dimos et al., 2008; Boulting et al., 2011; Bilican et al., 2012; Egawa et al., 2012). Importantly, iPSCs could be derived from older individuals, and the mutations in ALS-associated genes did not impair their ability to differentiate into MNs (Dimos et al., 2008). MNs with *TDP-43* mutations displayed cytosolic aggregates and decreased survival *in vitro*, in particular upon addition of a cellular stressor, such as sodium arsenite that induces oxidative stress (Bilican et al., 2012; Egawa et al., 2012).

**RESOURCE IMPACT**

**Background**

Amyotrophic lateral sclerosis (ALS) is a fatal neurodegenerative disease caused by loss of motoneurons, leading to progressive muscle atrophy. The majority of cases are sporadic; however, several genes have been recently linked to ALS, including the fused in sarcoma/translocated in liposarcoma (*FUS*) gene. Whereas wild-type FUS protein is predominantly nuclear, ALS-associated mutations cause its cytoplasmic delocalization. FUS cytoplasmic inclusions are found in the brain and spinal cord of individuals with ALS. However, the link between *FUS* mutations and motoneuron death is currently missing. In rodent models, overexpression of either mutant or wild-type *FUS* seems to exert similar outcomes, pointing to a general detrimental consequence of increased protein levels rather than a specific effect of protein mutations as the cause of motoneuron death in these models. *In vitro* studies have been mainly carried out in cancer cell lines that ectopically express mutant or wild-type proteins. These cell systems do not recapitulate the complexity of the motoneuron and its microenvironment and/or imply non-physiological levels of protein, which has hampered an accurate molecular analysis of ALS pathogenesis.

**Results**

In this study, the authors take advantage of two powerful technologies to develop a novel cell system to study the pathological mechanisms underlying FUS-linked ALS. First, they generate patient-specific induced pluripotent stem cells (PS-iPSCs), which carry the same FUS mutations found in ALS-affected individuals and can be differentiated into motoneurons. Then, by developing and optimizing a protocol of gene editing – a methodology that allows the introduction of specific changes in a cell's genome – the authors generate additional FUS-mutant iPSC lines that carry a severe mutation associated with juvenile ALS. By using these new *in vitro* models, the authors show that the incorporation of mutated FUS into cytoplasmic stress granules occurs in both undifferentiated iPSCs and iPSC-derived motoneurons when these are subject to various ALS-related cellular stressors (including oxidative stress).

**Implications and future directions**

The iPSC-based *in vitro* model described here allows the evaluation of the effects of patient-specific ALS-associated FUS mutations on ALS-relevant cell types, e.g. motoneurons. It also helped to evaluate the relevance of stress components in the pathogenesis of ALS. In addition, these cell lines, derived from patient-cell reprogramming or generated by site-directed mutagenesis, represent the ideal platform to dissect the molecular and cellular defects downstream of FUS mutations that could contribute to motoneuron death. Moreover, they will be instrumental for *in vitro* drug screening, as previously shown with analogous iPSC-based systems for other pathologies.

Incorporation of FUS into SGs in response to stress has been studied mostly in cell lines ectopically expressing mutant or wild-type (WT) proteins (Gal et al., 2011; Dormann et al., 2010, 2012; Bosco et al., 2010; Ito et al., 2010; Vance et al., 2013; Baron et al., 2013; Shelkovnikova et al., 2013), or, more recently, in ALS patients' fibroblasts (Vance et al., 2013). In this study, we aimed at generating a cell system, which would be more relevant to the pathology and in which FUS mutants are expressed endogenously at physiological levels. We generated iPSC lines from two ALS patients with *FUS* mutations, along with two controls: one from a healthy individual, devoid of mutations in *FUS* and *TDP-43*, and one from an age-matched patient with a mutation in *TDP-43*, to serve as a non-FUS ALS iPSC line. By TALEN-directed mutagenesis we also produced two additional mutant lines, carrying in homozygosis or heterozygosis the *FUS* P525L mutation associated with a severe and juvenile ALS form. We show here that different kinds of stress, including oxidative, heat and osmotic stress, induce preferential recruitment of mutant FUS in SGs both in undifferentiated iPSCs and differentiated ventral spinal cord neural populations, including MNs. Levels of FUS within SGs correlated with the degree of cytoplasmic delocalization of mutant proteins in unstressed conditions.

## RESULTS

### Generation of iPSCs from ALS patients’ fibroblasts

ALS patients harboring mutations in the *FUS* or *TDP-43* genes were recruited in order to obtain skin biopsies. Informed donors included three individuals with different heterozygous mutations in *FUS*, R514S (ALS I–FUS^R514S/wt^; age 49; female), R521C (ALS II–FUS^R521C/wt^; age 39; male) (Chiò et al., 2011) and P525L (ALS IV–FUS^P525L/wt^; age 20; female), and one individual with a homozygous A382T mutation in the *TDP-43* gene (ALS III–FUS^A382T/A382T^; age 50; male) (Borghero et al., 2011).

From the biopsies we derived primary dermal fibroblasts. Fibroblasts from a healthy individual (age 8), devoid of mutations in *FUS* and *TDP-43* (supplementary material Fig. S1), served as control (WT I). Patients and control cells were reprogrammed into iPSCs by taking advantage of a single lentiviral vector constitutively expressing the four human reprogramming factors, OCT4 (POU5F1 – Human Gene Nomenclature Database), KLF4, SOX2 and cMYC (hSTEMCCA) (Somers et al., 2010). iPSC-like colonies were obtained from control and ALS I, ALS II and ALS III fibroblasts, but not from ALS IV (see Materials and Methods for details).

**Fig. 1.**
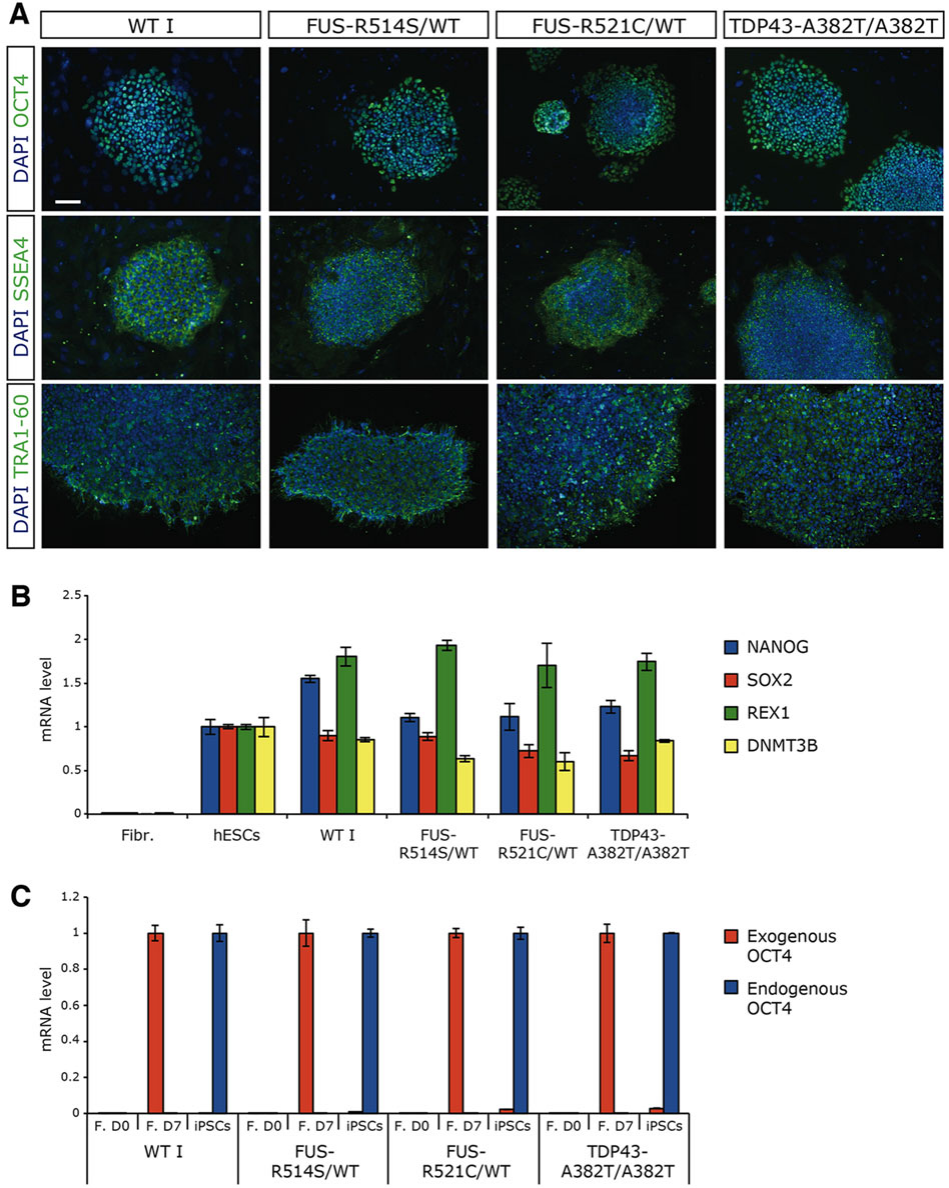
**Generation of iPSC lines and ventral spinal cord differentiation.** (A) Immunostaining of the pluripotency markers OCT4 (top panels), SSEA4 (middle panels) and TRA1-60 (bottom panels) in iPSCs derived from a healthy donor (WT I) or three ALS patients carrying the mutations in *FUS* or *TDP-43* indicated above each panel. Nuclei are counterstained with DAPI. Shown here are representative images for WT I-clone 2; ALS I-clone 2; ALS II-clone 34; ALS III-clone 2. Scale bar for all panels: 100 μm. (B) RT-qPCR analysis of the indicated pluripotency markers in fibroblasts (WT I), hESCs (RUES2 line) and iPSC clones. Levels of mRNA are normalized to those of hESCs. (C) RT-qPCR analysis of the expression of endogenous and exogenous (i.e. carried by the reprogramming vector hSTEMCCA) OCT4 in untreated fibroblasts (F. D0), fibroblasts after 7 days of infection with hSTEMCCA (F. D7) and iPSC clones. Levels of endogenous OCT4 mRNA are normalized to those of iPSCs, whereas levels of exogenous OCT4 mRNA are normalized to those of F. D7. In B and C, representative analyses for WT I-clone 5, ALS I-clone 1, ALS II-clone 34 and ALS III-clone 2 are reported. Similar analyses for other clones are shown in supplementary material Fig. S1.

After reprogramming, single iPSC-like colonies with uniform flat morphology and defined borders were selected for expansion as individual clones. Several clones per line were then validated as bona fide iPSCs. Expression of pluripotency markers was assessed by immunostaining (OCT4, SSEA4 and TRA1-60; Fig. 1A) and by real-time RT-PCR (RT-qPCR) (NANOG, REX1, SOX2, DNMT3B; Fig. 1B and supplementary material Fig. S1A). We also confirmed that expression of the exogenous reprogramming factor gene *OCT4* was silenced upon reprogramming, with a corresponding upregulation of its endogenous counterpart (Fig. 1C and supplementary material Fig. S1B). Pluripotency of the new iPSC lines was verified by multi-lineage differentiation assays *in vitro*. RT-PCR analysis showed induction of ectoderm, mesoderm and endoderm markers (supplementary material Fig. S1C). Finally, sequencing of *FUS* and *TDP-43* confirmed the presence of the expected mutations in iPSC lines derived from the respective patients (supplementary material Fig. S1D). We also checked for the absence of mutations in the same genomic regions of the *FUS* and *TDP-43* genes in the iPSC line derived from the control donor (iPSC-WT I; supplementary material Fig. S1E,F).

Taken together, this analysis confirmed that we successfully reprogrammed somatic cells from patients with mutations in *FUS* and *TDP-43*, generating bona fide patient-specific iPSCs (ALS-iPSCs).

**Fig. 2.**
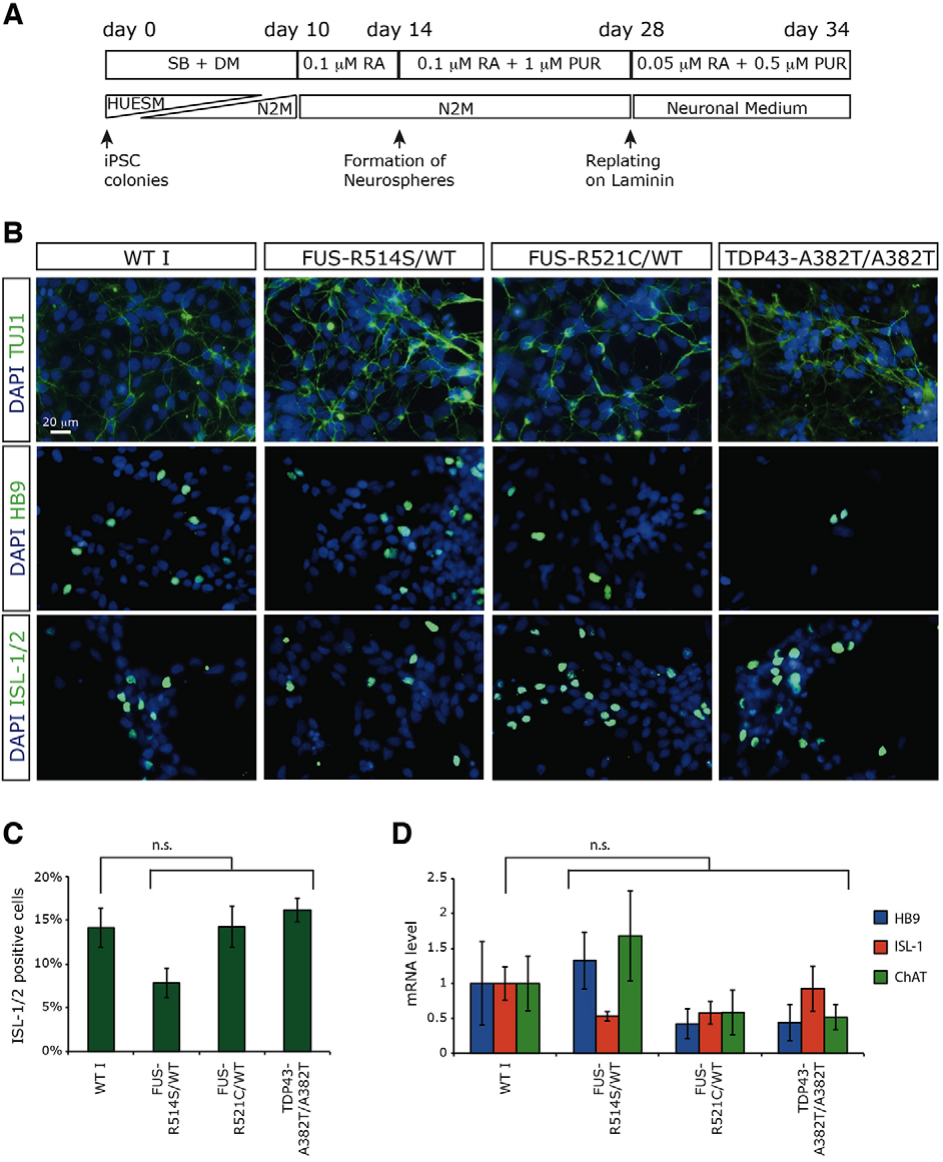
**MN differentiation.** (A) Schematic representation of the differentiation protocol. SB, SB431542 (Nodal/Activin inhibitor); DM, dorsomorphin (BMP inhibitor); RA, all-trans retinoic acid; PUR, purmorphamine (SHH agonist); HUESM, differentiation medium; N2M neural differentiation medium. (B) Immunostaining for the pan-neuronal marker TUJ1 (top panels) and the MN markers HB9 (middle panels) and ISL1/2 (bottom panels) in control and ALS iPSCs differentiated for 34 days. Nuclei are counterstained with DAPI. Scale bar for all panels: 20 μm. (C) Percentage of MN marker ISL1/2-positive cells detected by immunostaining analysis in differentiated iPSCs (34 days). Error bars represent s.d. Statistical analysis (Student's *t*-test) showed no significant difference in the differentiation ability of mutant iPSCs compared with the WT I line. (D) RT-qPCR analysis of the MN markers HB9, ISL1 and CHAT (choline O-acetyltransferase) in control and ALS iPSCs differentiated for 34 days. Histogram bars represent the averages from six (WT I, ALS I and ALS II) or four (ALS III) independent differentiations. Error bars represent s.e.m. Statistical analysis (Student's *t*-test) showed no significant difference in the differentiation ability of mutant iPSCs compared with the WT I line.

In order to differentiate ALS-iPSCs into spinal cord populations containing MNs, we adapted a previously established differentiation protocol that includes an initial phase of neural induction followed by the regional specification by retinoic acid (RA) and sonic hedgehog (SHH) (Wichterle et al., 2002; Hu and Zhang, 2010). Initial differentiation along the neuroectodermal lineage was triggered by dual-SMAD signaling inhibition in feeder-free iPSC cultures (Fig. 2A). This was followed by patterning with RA and detachment of neural precursors that were cultured as floating neurospheres. Further patterning with purmorphamine (PUR), an SHH agonist, and replating on laminin-coated plates led to differentiation of neuronal cells (Fig. 2B and supplementary material Fig. S2A). A subset of these neural cells expressed the specific MN markers HB9 (MNX1) and ISL1/2 (Fig. 2B-D and supplementary material Fig. S2B,C). As expected for a ubiquitous gene, *FUS* levels did not change upon differentiation (supplementary material Fig. S2D). Analysis of the expression of the specific markers OLIG2, HB9, ISL1 and CHAT in WT I and ALS iPSCs suggested that there is no impairment of MN differentiation in mutated lines (Fig. 2C,D and supplementary material Fig. S2C), as previously described for SOD1 and other TDP-43 mutants (Dimos et al., 2008; Bilican et al., 2012; Egawa et al., 2012).

### Generation of a mutant iPSC FUS^P525L^ line by TALEN-directed mutagenesis

**Fig. 3.**
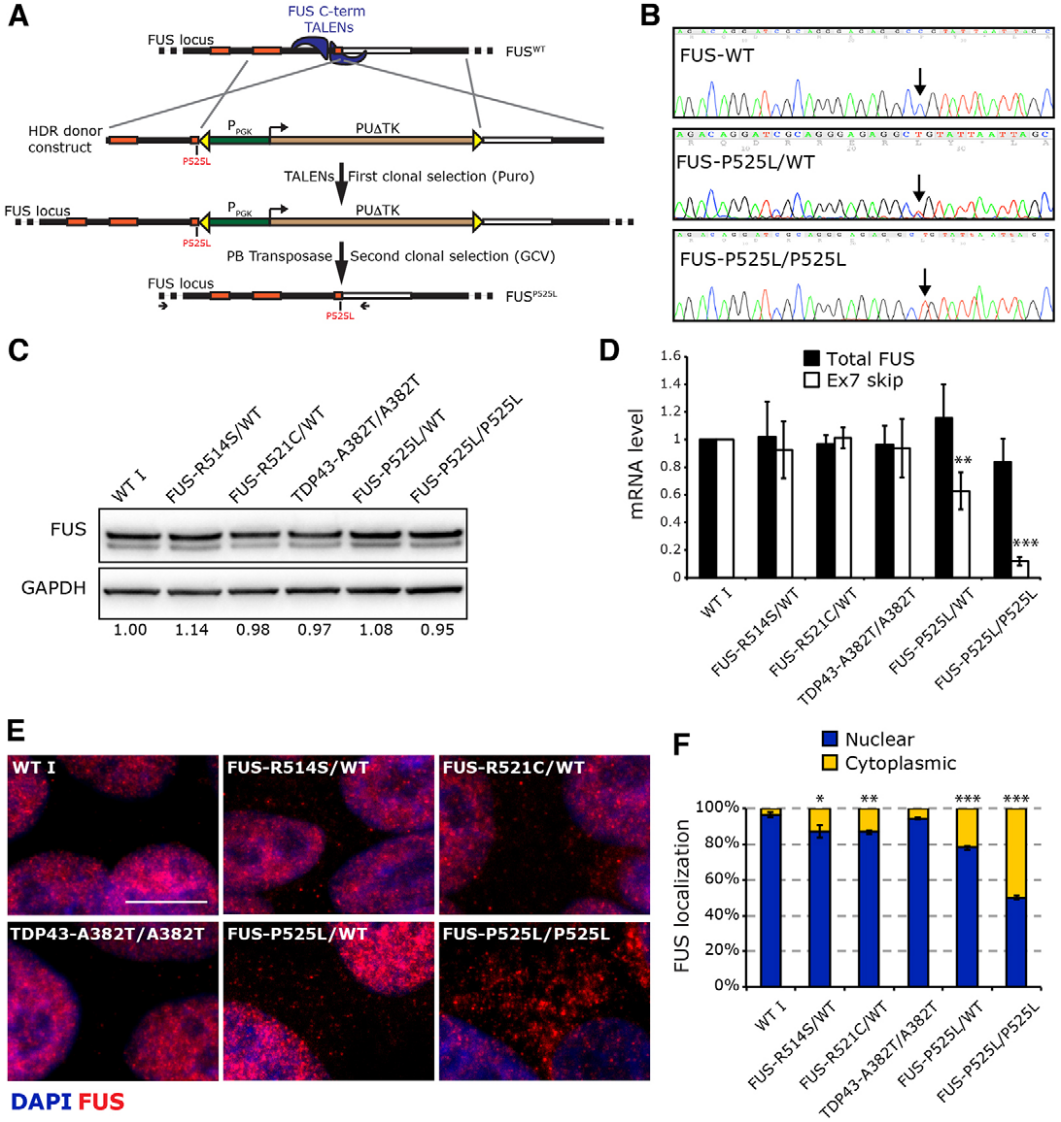
**Generation of FUS^P525L^ iPSC lines by TALEN-directed mutagenesis.** (A) Schematic of the TALEN/piggyBac combined strategy to generate the FUS^P525L^ mutant iPSC lines. On the top, the WT FUS locus and TALENs are depicted. Below, the HDR donor construct is schematized. P_PGK_, phosphoglycerate kinase 1 promoter; PUΔTK, fusion between PuroR and DeltaTK (truncated version of HSV type 1 thymidine kinase). Yellow triangles represent enhanced piggyBac (ePB) terminal repeats. The P525L mutation is indicated in red. The expected product of the homologous recombination and the FUS locus after PB-mediated excision is shown at the bottom. Horizontal arrows indicate primers used for PCR amplification from genomic DNA of the fragments sequenced in B. (B) Sequencing results from WT I iPSCs (top), iPSCs modified by TALEN-directed HDR after removal of the selection cassette (middle, heterozygous; bottom, homozygous). The arrows indicate the targeted nucleotide in codon 525 (C, wild type; T, mutant). Further details are provided in supplementary material Figs S3-S6. (C) Western blot analysis of FUS protein levels in iPSC lines used in this study. GAPDH is used as loading control. Densitometric quantification of FUS protein, relative to WT I, is shown below. (D) RT-qPCR analysis of FUS total mRNA and alternatively spliced mRNA devoid of exon 7 in iPSC lines. Quantification is relative to the levels in the WT I sample. (E) Immunostaining showing intracellular localization of WT and mutant FUS proteins in iPSCs. Scale bar: 10 μm. (F) Quantification of the immunostaining signal, showing FUS intracellular distribution in iPSC lines. In D and F, significant difference from the WT I was assessed by unpaired Student's *t*-test; *P<0.05, **P<0.01, ***P<0.001.

It is known that FUS mutations associated with severe forms of ALS localize in the C-terminal domain of the protein that contains the nuclear localization signal (Dormann et al., 2010). Whereas ALS I (FUS^R514S/wt^) and ALS II (FUS^R521C/wt^) patients developed the disease in mid-late age, the ALS IV (FUS^P525L/wt^) patient displayed a juvenile onset of the disease. As we did not manage to obtain iPSCs from FUS^P525L/wt^ fibroblasts, we generated iPSC lines with this mutation by genome editing. Our strategy, based on TALENs, allowed us to introduce the P525L mutation in the endogenous FUS locus of the iPSC-WT I line. The design, validation and optimization of the TALEN pair specific for FUS exon 15 (FUS C-term TALENs) is shown in supplementary material Figs S3-S5. We took advantage of the FUS C-term TALENs to produce iPSC FUS^P525L^ lines devoid of other genomic changes. The methodology, depicted in Fig. 3A, was based on a two-step positive/negative selection strategy. In the first step, homology-directed repair (HDR), stimulated by TALENs, promoted the insertion of a selection cassette flanked by the enhanced piggyBac (ePB) terminal repeats. The ePB is the humanized version of an insect transposon (piggyBac, PB) that transposes into a TTAA sequence, which is perfectly reconstituted upon excision, leaving no changes in the host genome after multiple transposition events (Lacoste et al., 2009). Thus, PB and its derivatives can be exploited as excisable cassettes, leaving no ‘scar’ in the host genome upon transposition (Yusa et al., 2011). The 5′ arm of the HDR donor construct included the P525L mutation, which is a C-to-T change in the second position of codon 525. The selection cassette contained an independent promoter (PGK) driving the expression of the PUΔTK bifunctional protein (Chen and Bradley, 2000), conferring resistance to puromycin and sensitivity to ganciclovir (GCV). After co-transfection with the FUS C-term TALENs, we isolated individual puromycin-resistant clones. Two of them contained the cassette inserted in one or both FUS alleles, thus providing heterozygous and homozygous situations, respectively (supplementary material Fig. S5). These clones were transfected with a modified PB transposase [hyPB int(−)], unable to re-integrate a sequence flanked by the PB terminal repeats (Li et al., 2013). In a preliminary optimization experiment, such hyPB int(−) resulted in the most efficient PB transposase for removing a selection cassette from host cells (supplementary material Fig. S4). After hyPB int(−) transient transfection, we counter-selected iPSCs retaining the exogenous cassette and isolated individual iPSC clones. Real-time PCR analysis on genomic DNA confirmed removal of the selection cassette in one sub-clone for each line (supplementary material Fig. S5A-E). PCR on genomic DNA confirmed that the selection cassette was not re-integrated in a silenced form elsewhere in the genome (supplementary material Fig. S5F). Before removal, the presence of the selection cassette did not significantly affect FUS protein levels (supplementary material Fig. S5G). To confirm the single nucleotide change in codon 525, the genomic region of interest was PCR-amplified and sequenced. As shown in Fig. 3B, genomic DNA sequencing revealed a double peak (C and T) in the heterozygous sub-clone and a single peak corresponding to the C-to-T nucleotide change in the homozygous sub-clone. Moreover, sequence analysis of the genomic region surrounding the target site (about 1 kb) revealed the correct reconstitution of the TTAA sequence after the PB transposase-mediated removal of the selection cassette and confirmed that the TALEN-induced HDR event did not introduce other genomic changes outside the mutated codon (supplementary material Fig. S6A). Finally, analysis of possible off-target effects of the TALENs confirmed the specificity of the utilized strategy (supplementary material methods).

In summary, the combination of site-directed mutagenesis by TALENs, followed by removal of the selection cassette by a modified PB transposase, allowed the generation of two novel iPSC lines, isogenic with our control iPSC line, containing the FUS^P525L^ mutation in heterozygosity or homozygosity. These new FUS^P525L^ mutant lines retained the ability to differentiate along the MN lineage with efficiency comparable to controls, ALS I and ALS II lines (supplementary material Fig. S2C).

As shown in Fig. 3C and D, the new FUS^P525L^ mutant iPSC lines expressed physiological levels of FUS protein and mRNA. Recent reports by our lab and others have shown that an autoregulatory feedback loop regulates FUS abundance (Zhou et al., 2013; Dini Modigliani et al., 2014). In particular, FUS can bind its own pre-mRNA and regulate alternative splicing of exon 7. Increased FUS levels trigger exon 7 skipping during the splicing reaction and production of an mRNA containing a premature termination codon, which undergoes nonsense-mediated decay (NMD). According to this model, a decrease in the nuclear levels of FUS should result in a reduction of the NMD mRNA form. As shown in Fig. 3D, a strong decrease of exon 7 skipping was indeed detected in FUS^P525L/P525L^ iPSCs and an intermediate decrease was observed in FUS^P525L/wt^, suggestive of a direct correlation between the amount of skipping and of nuclear FUS. The exon 7-devoid isoform represents a minor fraction (1-2%) of total FUS mRNA (Dini Modigliani et al., 2014). Thus, further reduction of this NMD isoform is not expected to significantly affect FUS protein levels. Furthermore, other mechanisms, such as repression by miR-200 (Dini Modigliani et al., 2014) that is abundantly expressed in iPSCs (data not shown), can blunt FUS protein levels.

**Fig. 4.**
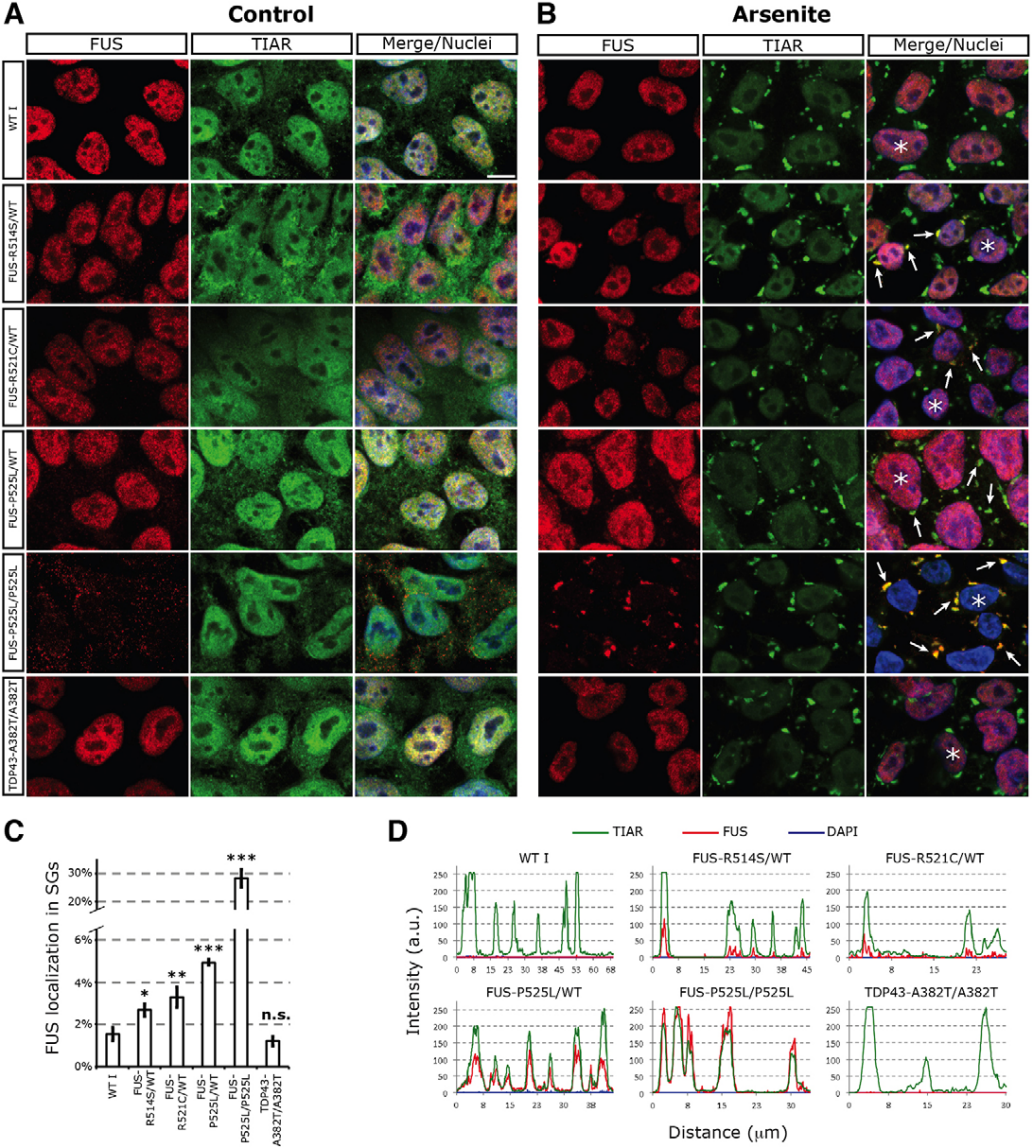
**Confocal analysis of FUS localization in control and stressed undifferentiated iPSCs.** (A,B) Immunostaining of FUS (red) and the SG marker TIAR (green) in undifferentiated iPSCs in control (vehicle-treated) conditions (A) or upon 0.5 mM sodium arsenite-induced oxidative stress for 60 min (B). A variable number of TIAR^+^ SGs could be observed in individual cells for all cell lines. Merge/nuclei panels show the combined signals of FUS, TIAR and DAPI. Scale bar: 10 μm. Arrows indicate examples of co-localization of TIAR and FUS signals in the cytoplasm. (C) Quantification of the immunostaining, showing the percentage of FUS signal in SGs. Statistically significant differences from WT I are indicated by asterisks (unpaired Student's *t*-test; *P<0.05, **P<0.01, ***P<0.001). (D) Representative linescan analysis of the TIAR, FUS and DAPI signal intensity in SGs of the cells indicated by asterisks in B. The line drawn across SGs and further analyses are shown in supplementary material Fig. S8.

**Fig. 5.**
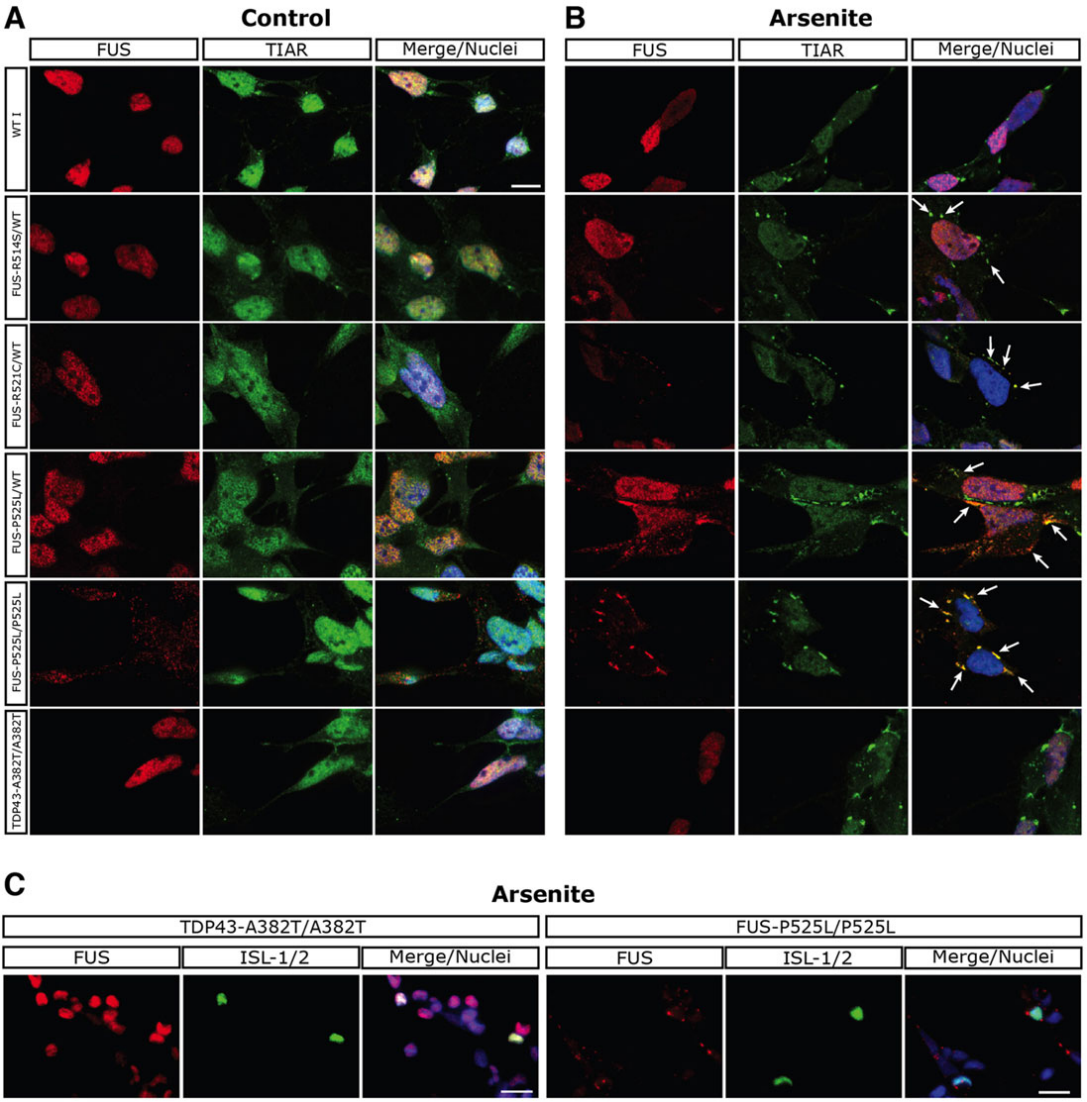
**Confocal analysis of FUS localization in control and stressed ventral spinal cord iPSC-derived cells.** (A,B) Immunostaining of FUS (red) and the SG marker TIAR (green) in iPSCs differentiated for 34 days, as in Fig. 2. (A) Control (vehicle-treated) conditions. (B) Oxidative stress induced by 0.5 mM sodium arsenite for 90 min. Merge/nuclei panels show the combined signals of FUS, TIAR and DAPI. Arrows indicate examples of co-localization of TIAR and FUS signals in the cytoplasm. (C) Immunostaining of FUS (red) and the MN marker ISL1/2 (green) in iPSCs differentiated for 34 days and stressed with 0.5 mM sodium arsenite for 90 min. Merge/nuclei panels show the combined signals of FUS, ISL1/2 and DAPI. Scale bars: 10 μm in A,B; 20 μm in C.

FUS intracellular distribution was analyzed by immunostaining. FUS^P525L/wt^ showed a strong protein delocalization with a punctuate cytoplasmic patterning (Fig. 3E and Fig. 4A). An even higher delocalization was detected in the FUS^P525L/P525L^ line. On the contrary, the iPSC lines carrying the FUS^R514S^ and FUS^R521C^ mutations showed a predominant FUS nuclear localization in both undifferentiated iPSCs (Fig. 3E and Fig. 4A) and in iPSC-derived ventral spinal cord cells (Fig. 5A), with only a minute cytoplasmic delocalization (Fig. 3E). Quantification of the immunostaining signals and cell fractionation indicated significant differences of FUS cytoplasmic levels between controls and mutants, as well as between strong and weak mutations (Fig. 3F and supplementary material Fig. S6B).

Altogether, these data show that in genetic backgrounds corresponding to those found in patients there is a progressive increase in cytoplasmic FUS accumulation from the R514S and R521C mutations to the P525L. Notably, in the heterozygous line FUS^P525L/wt^ the amount of cytoplasmic FUS was reduced by half compared with FUS^P525L/P525L^ (Fig. 3E,F).

### Endogenous FUS mutants display altered recruitment in SGs in undifferentiated and differentiated iPSCs

Several kinds of cellular stress have been shown to induce ectopically expressed mutant FUS protein recruitment into cytoplasmic SGs (Bentmann et al., 2012). The re-localization of mutant and WT FUS upon stress in undifferentiated iPSCs was first tested with sodium arsenite (ARS) to mimic oxidative stress. Immunostaining, analyzed by confocal microscopy, showed that the iPSC-WT I line displays nuclear FUS localization both in the absence (Fig. 4A) and in the presence of ARS (Fig. 4B). In unstressed ALS I-FUS^R514S/wt^ and ALS II-FUS^R521C/wt^ cells, the FUS signal is mainly nuclear, and upon ARS treatment only few cytoplasmic speckles are visible (Fig. 4A,B). In heterozygous FUS^P525L/wt^ cells, a greater number of FUS^+^ speckles were induced by ARS (Fig. 4B). Co-localization with the SG markers TIAR (Fig. 4B) and PABP (supplementary material Fig. S7) indicated the specific localization of mutant FUS in these structures. As the antibody cannot distinguish between WT and mutant FUS, it is impossible to establish whether only the mutant FUS protein is present in SGs or whether the normal protein also co-localizes inside the granules. Notably, in homozygous FUS^P525L/P525L^ iPSCs, whereas a strong and punctuate cytoplasmic delocalization of FUS is observed in control conditions, upon ARS treatment the majority of cytoplasmic signals concentrate into SGs (Fig. 4A,B). Conversely, FUS remained nuclear restricted in stressed ALS III iPSCs. These cells, carrying the TDP-43 mutation, serve as a further control of a non-FUS ALS mutation. Quantification of the FUS signal upon oxidative stress confirmed the accumulation of higher protein levels within SGs in mutants compared with controls (Fig. 4C). In the different mutants, the fraction of FUS localized in SGs correlated with the degree of cytoplasmic delocalization observed in unstressed cells (Fig. 3F). A linescan analysis showed FUS localization, even if at low levels, in most of the SGs induced by ARS in the weak mutants (FUS^R514S^ and FUS^R521C^) (Fig. 4D and supplementary material Fig. S8). The severe P525L mutation caused much higher levels of FUS recruitment, which was detectable in all SGs analyzed (Fig. 4D and supplementary material Fig. S8). Taken together, these analyses showed that the severity of the mutation, measured by the delocalization phenotype in unstressed conditions, correlates with the ability of endogenous FUS proteins to engage in SGs upon stress.

We next assessed whether recruitment into SGs of endogenous FUS mutants was reversible and reproducible with different kinds of stress. Upon removal of ARS and recovery in normal medium for three hours, SGs were dissolved both in WT and mutant cells (supplementary material Fig. S9). However, the kinetics of SGs dissolution could be affected by the presence of mutant FUS, as FUS^P525L/wt^ cells displayed a greater number of SGs than WT cells at earlier time points of recovery (supplementary material Fig. S9). Heat shock, caused by a raise of the temperature to 44°C for 1 h, caused SGs localization of the FUS mutant proteins, but not of WT or ALS III (supplementary material Fig. S10). In the case of hyperosmolar stress induced by sorbitol treatment (Sama et al., 2013), we observed increased cytoplasmic FUS delocalization in the mutants and, to a minor extent and at later time points, also in the WT and ALS III (supplementary material Fig. S11). In this analysis, we could not proceed beyond 25 min of sorbitol exposure, as iPSCs survival was severely compromised after this time point. At 25 min, SGs are not yet fully formed and the most evident effect of this stress condition was on the cytoplasmic localization of FUS rather than on its recruitment into SGs.

Overall, these data suggest the hypothesis that the ability to form FUS^+^ SGs directly depends on the amount of cytoplasmic delocalized FUS.

Finally, we studied FUS localization in WT and mutant lines after differentiation to ventral spinal cord neural cells for 34 days (Fig. 2), in control conditions and during stress. Our analysis confirmed SG recruitment of endogenous FUS in the four mutants upon oxidative, temperature and hyperosmolar stress (Fig. 5 and supplementary material Figs S12-S16). In neural cells, similarly to what we observed in undifferentiated iPSCs, the ability to recruit FUS into SGs correlated with the amount of cytoplasmic delocalized protein in unstressed conditions. In particular, the highly delocalized FUS^P525L^ mutant was detected in almost all TIAR^+^ granules in both FUS^P525L/wt^ and FUS^P525L/P525L^ lines (Fig. 5 and supplementary material Fig. S16). Co-staining with the specific marker ISL1/2 allowed us to visualize specifically MNs; notably, also in these cells FUS^+^ SGs were present in the mutant, but not in the control (Fig. 5C). In this analysis, we did not observe any difference in FUS ability to aggregate in SGs in MNs versus surrounding neurons (Fig. 5C). In WT differentiated cells, FUS recruitment was observed in SGs only upon hyperosmolar stress (supplementary material Fig. S15). Notably, in sorbitol-treated cells, a striking difference between the FUS mutants and the WT was observed in the amount of nuclear-localized FUS protein. Compared with WT cells, nuclear FUS was strongly depleted in all FUS mutants (supplementary material Fig. S15), suggesting a peculiar activity of this stress stimulus in increasing cytoplasmic accumulation also of those FUS mutants with a weak delocalization phenotype. In the TDP-43 mutant ALS III line, the localization of endogenous FUS closely mirrored the WT control in all conditions tested.

In conclusion, our analysis showed preferential recruitment of endogenous mutant FUS in SGs, in both proliferating iPSCs and differentiated MNs. Upon exposure to oxidative or temperature stress, FUS localization into SGs occurred exclusively in mutant lines, although to different extents.

## DISCUSSION

Currently, several *in vivo* systems of ALS have been produced, including rodents, *Drosophila melanogaster* and *Caenorhabditis elegans* (Shelkovnikova, 2013). Transgenic mouse models of ALS display dose-dependent toxicity of FUS and TDP-43, usually expressed under neuronal-specific promoters (McGoldrick et al., 2013). Notably, overexpression of either mutant or wild-type proteins seems to exert similar outcomes, pointing to a general detrimental consequence of the alteration of protein levels, rather than a specific effect of the mutations present in ALS patients, as the cause of MN death in these models. Therefore, the ALS pathology might be caused not only by mutations that alter the structure of disease-associated proteins, but also by their altered levels. This hypothesis is further supported by the recent finding of mutations associated with severe forms of ALS not affecting the FUS coding sequence but rather the 3′-UTR regulatory sequences (Sabatelli et al., 2013). We recently showed that one of these ALS-associated mutations disrupts an miRNA binding site, producing increased levels of otherwise normal FUS protein (Dini Modigliani et al., 2014). On the other hand, most *in vitro* studies with human cells rely on non-neural or neuroblastoma cell lines, in which mutated FUS or TDP-43 genes were overexpressed. Such systems do not recapitulate the complexity of the motoneuron and its microenvironment and imply non-physiological levels of protein.

In this study, we have successfully reprogrammed fibroblasts from two ALS patients carrying the most common FUS mutations (FUS^R514S^ and FUS^R521C^). iPSCs from these patients can be differentiated into cell types relevant for the disease, representing a useful tool to model the ALS pathology *in vitro*. Moreover, as fibroblasts from another severe FUS mutation (P525L) were not available, we made an effort in producing and optimizing a TALEN-based approach to mutate the endogenous *FUS* gene in control iPSCs. Our strategy was based on a two-step protocol that combines HDR triggered by TALENs with seamless removal of the selection cassette by an improved PB system [ePB terminal repeats used in combination with the hyperactive int(−) transposase]. Importantly, with the tools described here virtually any desired mutation can be inserted in any gene of interest. Analysis of specific markers suggested that WT and FUS mutant iPSCs can be differentiated into MNs with comparable efficiency, even though it cannot be excluded that some functional impairment in iPSC-derived MNs could occur. In the future, electrophysiological analysis on differentiated MNs will allow clarification of this aspect.

Most of ALS-linked FUS mutations are localized in the C-terminal domain of the protein, which contains the PY nuclear localization signal (Kwiatkowski et al., 2009; Gal et al., 2011). Ectopic expression of ALS-associated FUS mutants in cell lines showed an inverse correlation between the degree of cytoplasmic mislocalization and the age of ALS onset in patients bearing the corresponding mutations. Typically, mutations in codons 514 and 521 cause mild delocalization and are found in late-onset ALS, whereas mutations in codons 525 (or 522), which strongly impair nuclear import, are associated with juvenile ALS forms (Dormann et al., 2010, 2012; Ito et al., 2010). Disruption of transportin-mediated nuclear import, leading to mislocalization of FUS in the cytoplasm, has been proposed as a causing determinant of the ALS pathology (Vance et al., 2009; Dormann et al., 2010; Bosco et al., 2010; Ito et al., 2010). The study of ALS physiopathology has been tremendously advanced by the discovery that both FUS and TDP-43 are abnormally deposited in neuronal and glial cytoplasmic inclusions in the majority of ALS and FTLD patients. A number of recent studies have indeed shown that some FUS mutants, ectopically expressed in cell lines or in primary rodent neurons, are recruited in SGs (Dormann et al., 2010; Bosco et al., 2010; Ito et al., 2010; Bentmann et al., 2012; Baron et al., 2013), which are cytoplasmic non-membrane-covered mRNP particles composed of poly(A)^+^ mRNAs and RNA-binding proteins. On the other hand, some SG markers have been found within pathological, FUS^+^, cytoplasmic inclusions in ALS patients (Dormann et al., 2010; Bäumer et al., 2010). These findings led to a model in which a nuclear import defect, due to mutations in the NLS, causes FUS recruitment into SGs, which, over time, might turn into pathological inclusions, probably due to increased local concentration and/or misfolding of FUS (Bentmann et al., 2013). An alternative model considers SGs as protective structures that prevent FUS aggregation in the cytoplasm. Over time, high levels of FUS expression would overcome such protective function and result in the formation of pathological inclusions (Shelkovnikova, 2013). Both models need further experimental validation in appropriate experimental systems. A crucial parameter to take into consideration is the amount of FUS protein. When mutant FUS is overexpressed by transient transfection, recruitment into SGs occurs in the absence of external stress (Gal et al., 2011; Ito et al., 2010). However, FUS mutants expressed by stably integrated inducible constructs assemble into SGs only upon exposure to stressors (Bosco et al., 2010). In these cells, lower levels of protein accumulation might be more representative of the physiological situation. Recently, such concentration-dependent behavior of mutant FUS has been better characterized by correlating intracellular localization with levels of ectopic expression. In particular, after reaching a given concentration threshold, diffuse cytoplasmic mutant FUS progressively aggregated, forming so-called FUS granules (FGs) that subsequently assembled into FUS aggregates (FAs) (Shelkovnikova et al., 2014). This evidence could represent an important clue to understanding the formation of pathological FUS inclusion during the progression of ALS, and it implies that the study of pathological FUS should be carried out in cell model systems expressing physiological levels of mutated proteins. To this aim, a recent report used skin fibroblasts from ALS patients to study mutant FUS incorporation in SGs (Vance et al., 2013). Interestingly, mutant FUS sequestration in nuclear aggregates has been also recently reported in patients' fibroblasts (Schwartz et al., 2014). However, despite FUS being ubiquitously expressed, fibroblasts and most other cell types are spared by ALS, which instead specifically affects MNs and their microenvironment. Therefore, confirmation in neural cell systems such as those described here would greatly substantiate observations made in fibroblasts.

Here, we took advantage of human iPSCs and their ventral spinal neural derivatives to study the response to stress of different endogenous FUS mutants expressed at physiological levels. We showed that in normal growth conditions the FUS^R514S^ and FUS^R521C^ mutant proteins were only minimally delocalized, whereas FUS^P525L^ was mostly cytoplasmic, with a punctuate distribution in this compartment. Instead, when stress signals were imposed on the cells, in all three cases the mutant proteins, with different efficiency, depending on their delocalization phenotype, were readily recruited into SGs. In particular, FUS in FUS^R514S/wt^ and FUS^R521C/wt^ lines has a milder SG localization phenotype compared with the FUS^P525L/wt^, which is typical of juvenile ALS. In line with these data, the most severe phenotype was provided by the FUS^P525L/P525L^ line, in which FUS had the highest cytoplasmic delocalization rate.

Notably, the wild-type protein markedly associated to SG only in very extreme stress conditions, represented by long exposure to hyperosmolar stress. In this case, however, a clear difference in SG recruitment and intracellular localization of mutant and WT FUS could be detected. These data indicate that in the genetic background of the mutants, the amount of delocalized protein is not able per se to induce SG recruitment that instead is produced only upon a stress stimulus.

Our data suggest the relevance of stress components in the ethiopathogenesis of ALS. Historically, oxidative stress has been proposed as a potential pathogenic factor in ALS since the discovery of mutations in the SOD1 gene, encoding for an antioxidant enzyme, in fALS patients (Renton et al., 2014). More recently, other kinds of stress have been also associated with the pathology. A compound that has the ability to induce heat-shock response is currently under investigation in a phase II clinical trial for ALS (Kalmar et al., 2014). Moreover, it has been recently shown that osmotic stress enhanced motoneuron degeneration in an animal model of ALS (Therrien et al., 2013). Therefore, all stress conditions tested in this work might be relevant for ALS.

As a future perspective, the iPSC-based *in vitro* model described here, derived from patients by reprogramming or generated by site-directed mutagenesis, might greatly improve our understanding of the molecular basis of MN death and possibly help finding new therapeutic targets for ALS. In particular, this model may be used to better characterize the mechanisms that lead these particular FUS mutant proteins to aggregate into SG. For instance, the analysis of MNs expressing physiological levels of mutated proteins will help to highlight irregularities in terms of SGs number, size and kinetics of aggregation/disassembly upon cessation of stress, and the effects of FUS mutations on general translation in normal conditions and under stress.

## MATERIALS AND METHODS

### Generation and maintenance of human iPSCs

For ALS I, II and III, skin biopsies of informed donor ALS patients have been collected in the Turin ALS center, Italy. Details on ALS II and ALS III cases can be found in Chiò et al. (2011) and Borghero et al. (2011), respectively. ALS I corresponds to case IV-7 in Chiò et al. (2009) (this patient was diagnosed with spinal-onset ALS after publication of that article). Dermal fibroblasts were generated from these explants and cultured in fibroblast basal medium (FBM; Lonza) containing 15% FBS, 1× L-Glu and 1× penicillin-streptomycin (all from Sigma-Aldrich). Whereas healthy cultures could be established from ALS I, II and III, growth of fibroblasts recovered from the ALS IV biopsy (kindly provided by Prof. M. Sabatelli, Catholic University, Rome, Italy) was strongly impaired. We hypothesize that reduced proliferation was due to contamination of the culture from adipose and epidermis tissue, which might prevent fibroblast growth. Control fibroblasts (WT I) from a healthy individual were kindly provided by Dr A. Musarò, Sapienza University of Rome, Italy. For reprogramming experiments, ALS and control fibroblasts in a 35 mm dish were infected in serum-free conditions and in the presence of 4 mg/ml polybrene with the lentiviral vector hSTEMCCA (Somers et al., 2010), carrying the four reprogramming factors OCT4, SOX2, KLF4 and cMYC in a single polycistronic unit. Seven days after infection, 50,000-100,000 fibroblasts were seeded on a feeder layer of mytomycin C-treated primary mouse embryonic fibroblasts (PMEF-CF; Millipore) in a 10 cm dish. The next day, the medium was changed with HUESM [DMEM-F12+glutamax, Life Technologies; 20% knockout serum replacement, Life Technologies; 1× non-essential amino acids (NEAA), Life Technologies; 1× penicillin-streptomycin, 0.1 mM β-mercaptoethanol, both from Gibco] supplemented with bFGF (10 ng/ml, BD Biosciences). Twenty days after infection, the medium was replaced with Nutristem-XF (Biological Industries). Around day 25, iPSC colonies were manually picked, fragmented and individually passaged in a 12-well plate (passage 0, p0) coated with PMEF-CF cells. We obtained iPSC colonies from ALS I, II, III and WT I fibroblasts. We failed to reprogram ALS IV fibroblasts, probably due to their poor proliferation rate in culture, as active cell division is necessary for reprogramming to occur. From each reprogramming experiment, typically 3-5 iPSC clones selected by morphology (flat and uniform colonies with defined borders) were expanded from p0. From p2-p3, established iPSC lines were maintained in Nutristem-XF in plates coated with hESC-qualified matrigel (BD Biosciences) and passaged every 4-5 days with 1 mg/ml dispase (Gibco). The analysis of pluripotency markers shown in Fig. 1A,B was performed at passage 2, 35-40 days after infection. For immunostaining analysis, undifferentiated iPSCs were plated in matrigel-coated μ-chamber 12-wells (Ibidi). Clones used in the experiments shown in Figs 2-5 were: WT I clone#1; ALS I clone#1; ALS II clone#34 and ALS III clone#2. These lines were used in parallel throughout the study. We did not detect significant differences between biological replicates, i.e. different iPSC clones derived from the same patient or control, tested in parallel for selected experiments (supplementary material Fig. S17).

### Differentiation of iPSCs into ventral spinal cord neural cells

iPSCs were passaged (passage number ∼10-20) in 35-mm plates, and after two days the medium was changed to HUESM supplemented with SMAD inhibitors (SB/DM; 10 μM SB431542 and 2.5 μM dorsomorphin, both from Miltenyi Biotec). This was considered day 0 (D0). From D4, in the presence of SB/DM, the medium was gradually replaced with N2M (DMEM-F12+glutamax; 1× N2 supplement, Life Technologies; 1× NEAA, Life Technologies; 2 μg/ml heparin, Sigma-Aldrich): at D4, 75% HUESM and 25% N2M; at D6, 50% HUESM and 50% N2M; at D8, 25% HUESM and 75% N2M. From D10 to D14, differentiating neural progenitors were cultured in N2M supplemented with 0.1 μM all-trans RA (Sigma-Aldrich). At D14, neural rosette fragments were manually detached to generate floating neurospheres, maintained in N2M (containing 1× B27, Life Technologies) supplemented with 0.1 μM RA and 1 μM purmorphamine (sc-202705, Santa Cruz). At D28, neurospheres were plated in poly-L-ornithine (Sigma-Aldrich) and natural mouse laminin- (Invitrogen) coated 35-mm dishes for RNA extraction, or in poly-L-lysine- (Sigma-Aldrich) and natural mouse laminin- (Invitrogen) coated glass coverslips for immunostaining, in N2M supplemented with 10% FBS. The day after, the medium was replaced with N2M supplemented with 10 ng/ml BDNF, 10 ng/ml GDNF and 10 ng/ml IGF (all from PreproTech), 1 μM cAMP and 200 ng/ml L-ascorbic acid (both from Sigma-Aldrich), 0.05 μM RA and 0.5 μM purmorphamine. Ventral spinal cord neural cells were collected for RNA or fixed for immunostaining at day 34.

### RT-PCR, RT-qPCR and western blot analyses

Total RNA was extracted with the RNeasy kit (Qiagen). For RT-qPCR and RT-PCR RNA was retrotranscribed with the SuperScriptIII kit (Invitrogen). As negative controls, minus-reverse transcriptase samples were included in subsequent amplification reactions (not shown). For RT-PCR, cDNA was used as template with the BioTaq DNA polymerase (Bioline). RT-qPCR analysis was performed with SYBR Green QPCR Master Mix (Qiagen) in a 7500 Fast Real-Time PCR System (Life Technologies) and calculations were performed with the delta Ct method. In RT-qPCR analyses, the internal control used was the housekeeping gene *ATP5O* (ATP synthase, H^+^ transporting, mitochondrial F1 complex, O subunit) for undifferentiated iPSCs, or *GAPDH* for differentiation experiments. A complete list of primers is provided in supplementary material Tables S1 and S2.

Western blot analysis of FUS protein levels was carried out with NuPAGE 4-12% Bis-Tris gels (Life Technologies) in MOPS-SDS buffer as in Morlando et al. (2012), using anti-FUS/TLS (Santa Cruz, sc-47711; 1:2000) and, as a loading control, anti-GAPDH (Santa Cruz, sc-32233; 1:3000) antibodies. Images were acquired with the Chemidoc MP (Bio-Rad) and protein levels quantified with the ImageLab software (Bio-Rad).

### Immunostaining and confocal imaging

Cells were fixed in 4% paraformaldehyde for 20 min at room temperature and washed twice with PBS. Fixed cells were then permeabilized with PBS containing 1% BSA and 0.2% Triton X-100 and incubated overnight with primary antibodies at 4°C. The primary antibodies used are: anti-OCT4 (BD Biosciences, 611202; 1:500), anti-SSEA4 (Abcam, ab16287; 1:80), anti-TRA1-60 (Life Technologies, 41-1000; 1:100), anti-FUS/TLS (Abcam, AB84078; 1:500), anti-HB9 (DSHB, 81.5C10; 1:100), anti-Islet-1/2 (DSHB, 39.4D5; 1:50), anti-TIAR (BD Biosciences, 610352; 1:1000), anti-PABP (Santa Cruz, SC-32318; 1:100) and anti-TUJ1 (Chemicon Mab, 1637; 1:100). The secondary antibodies used are: goat anti-mouse Alexa Fluor 488 (Invitrogen, A11029; 1:300), goat anti-mouse Cy3 (Jackson ImmunoResearch, 115-165-003; 1:600), goat anti-rabbit Alexa Fluor 488 (Invitrogen, A11008; 1:200), goat anti-rabbit DyLight 549 (Vector Laboratories, DI-1549-1.5; 1:300) and goat anti-rabbit Cy3 (Jackson ImmunoResearch, 111-165-003; 1:300). DAPI (Sigma-Aldrich) was used to label nuclei. For the immunostaining shown in Figs 1 and 2, as negative controls mouse embryonic fibroblasts (MEFs) were stained in parallel with iPSCs and showed no signal (data not shown). Cells in Figs 1 and 2 were imaged with an Axioscope (Zeiss) microscope. Confocal images in Figs 3-5 were acquired using an inverted Olympus iX73 microscope equipped with an X-light Nipkow spinning-disk head (Crest Optics) and Lumencor Spectra X LED illumination. Images were collected using a CoolSNAP MYO CCD camera (Photometrics) and MetaMorph software (Molecular Devices) with a 60× oil objective. For Fig. 5C a 20× objective was used. For each sample, image stacks (26 images of 0.2-μm *Z*-step size) were acquired for each wavelength. Images are shown as maximum-intensity projections of seven planes. To count ISL1/2-positive cells, we used the Count Nuclei App of the MetaMorph software, running the segmentation on the DAPI channel to estimate the total number of cells and on the ISL1/2 channel to estimate the total number of MNs. We counted 1000-6000 cells per line.

### Quantification of nuclear/cytoplasmic and SG FUS distribution and linescan analysis

All image analyses were performed with MetaMorph V7.8.0 software. Before quantification, background signal was subtracted by statistical correction for each FUS channel image. Resulting images were analyzed with the integrated morphometry analysis (IMA) tool. For nuclear/cytoplasmic distribution, a nuclear mask was generated in the DAPI channel and then used in the IMA tool to measure FUS intensity in the same nuclear region. Cytoplasmic FUS intensity was derived by subtracting the nuclear intensity from the total intensity obtained with the region measurements tool. This analysis was performed on at least 100 cells per line, excluding dividing cells. Quantification of FUS inside SGs was performed as follows: to generate a mask of the SGs we took advantage of PABP as SG marker (supplementary material Fig. S7) as it allowed for a more accurate segmentation of the SG signal than TIAR. We used the Count Nuclei App to segment the PABP channel and generate an SG mask. To avoid an overestimation of SG-FUS, the SGs overlapping the nucleus were eliminated using a nuclear mask (created as described before). The corrected SG mask was then used in the IMA tool to measure SG-FUS intensity. Total FUS intensity was measured as described before. This analysis was performed on at least 100 cells per line.

Linescan analysis was performed with the Linescan MetaMorph tool to measure and graph the intensity values along a selected line in a 24-bit color image. Data were graphed with separate traces for the red, green and blue components. We used the multi-line tool to draw a line encompassing multiple SGs.

### TALEN-directed mutagenesis

Cloning of TALENs constructs targeting FUS exon 15 (FUS C-term TALENs) was carried out following the Golden Gate strategy as described in Sanjana et al. (2012) (Addgene TALEN Kit #1000000019). Details on the TALEN constructs are provided in supplementary material methods. Donor constructs for homologous recombination have been generated by cloning a DNA fragment of 1130 bp, including the section of the FUS locus depicted in Fig. 3, in the pBluescript KS(−) plasmid (Stratagene). Starting from this construct, we generated the FUS-EGFP-T2A-PuroR donor (supplementary material Fig. S3) and the FUS-PB-PGK-PUΔTK donor (Fig. 3A). Oligonucleotides used for cloning are provided in supplementary material Table S3. FUS C-term TALENs (1.5 μg each) and donor constructs (2 μg) were co-transfected in iPSC-WTI cells (clone #1). All transfections were carried out as follows: iPSCs were treated for 1 h with 10 μM ROCK-Inhibitor (Y-27632, Sigma-Aldrich) to enhance survival, trypsinized, and electroporated with a Neon Transfection System (Life Technologies) with 100 μl tips in R buffer and the following settings: 1200 V, 30 ms, 1 pulse. After transfection, iPSCs were seeded and cultured in presence of 10 μM ROCK-Inhibitor for one day before switching to normal culture conditions. Selection was carried out with 0.5 μg/ml puromycin for 7 days, and surviving clones were individually passaged and characterized as described in supplementary material Figs S3-S5. To remove the PB-PGK-PUΔTK selection cassette, the cells were transfected with 5 μg of the hyPB int(−) piggyBac transposase. After 7 days, 2 μg/ml ganciclovir (Sigma/Aldrich) was added to the medium, and surviving clones were individually passaged and characterized as described in supplementary material Fig. S5. Genomic DNA was isolated with the genomic DNA extraction kit (RBC Biosciences) and the region surrounding FUS exon 15 was PCR-amplified with the primers depicted in Fig. 3. In particular, the forward primer annealed outside of the 5′ homology arm and the reverse primer in the 3′-UTR. Sequencing of the DNA amplicon was carried out by Bio-Fab Research.

## Supplementary Material

Supplementary Material
