# Supplementary material for: ALS mutant FUS proteins are recruited into stress granules in induced pluripotent stem cell-derived motoneurons
Source: Dis Model Mech. 2015 Jul 1;8(7):755–66. doi: 10.1242/dmm.020099 (PMC4486861; doi:10.1242/dmm.020099)
Supplement: Supplementary Material [file supp_8_7_755__index.html]

Supplementary Material 

# ALS mutant FUS proteins are recruited into stress granules in induced Pluripotent Stem Cells (iPSCs) derived motoneurons

## DMM020099 Supplementary Material

- Supplementary Material
