# Supplementary material for: ALS mutant FUS proteins are recruited into stress granules in induced pluripotent stem cell-derived motoneurons
Source: Dis Model Mech. 2015 Jul 1;8(7):755–66. doi: 10.1242/dmm.020099 (PMC4486861; doi:10.1242/dmm.020099)
Supplement: Supplementary Material [file supp_020099_DMM020099supp.pdf]

Lenzi et al., DMM 2015

SUPPLEMENTARY MATERIAL

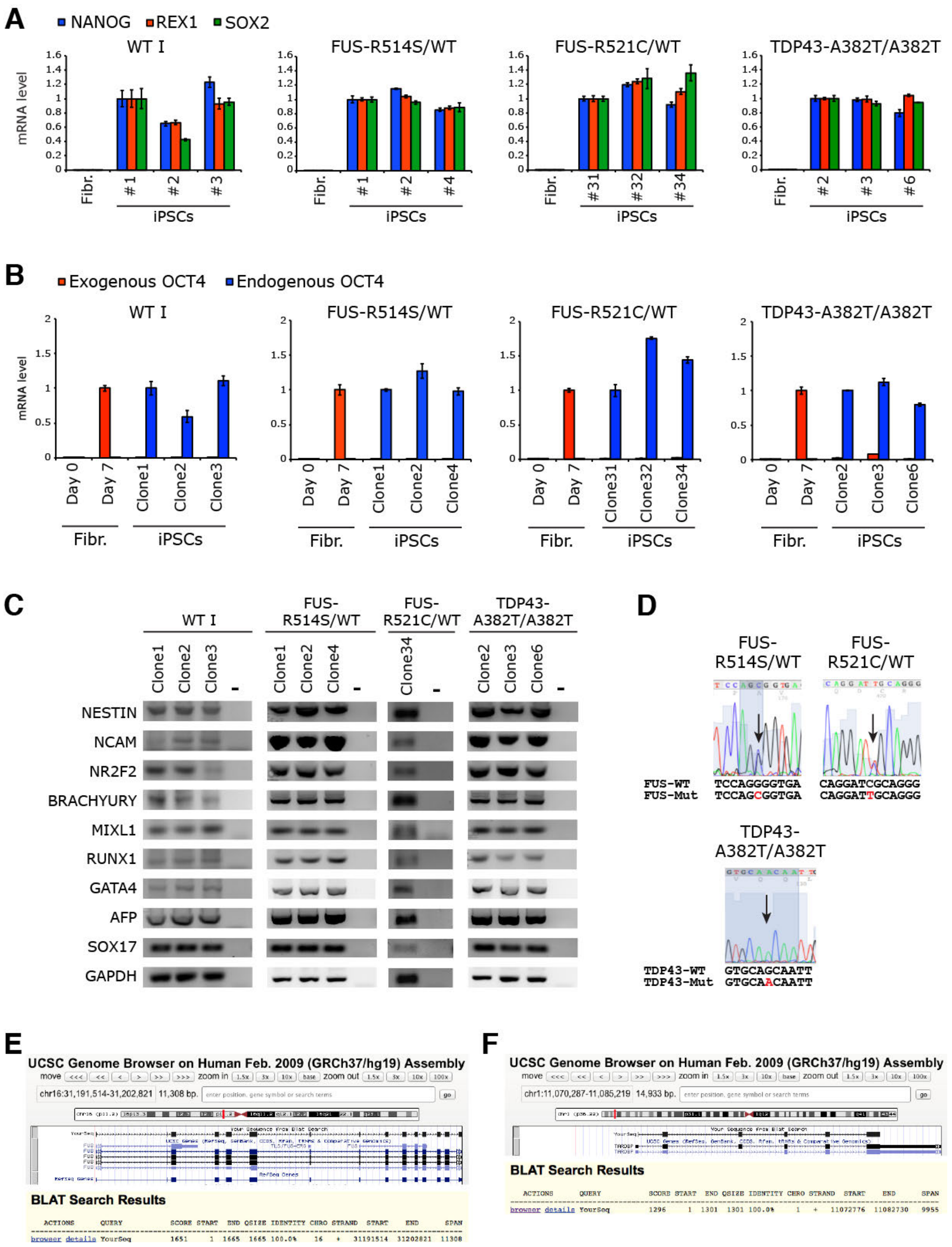

Figure S1 – Characterization of control and ALS patients-derived iPSCs.

(**A**) Real-time qRT-PCR analysis of the indicated pluripotency markers in fibroblasts and iPSCs. For each iPSC line, different clones (indicated with #) are analyzed here. (**B**) Real-time qRT-PCR showing expression of the exogenous OCT4 transgene, encoded by the reprogramming viral vector hSTEMCCA (red bars), and the endogenous OCT4 gene (blue bars), using specific primers. Different clones for each iPSC line are shown. Fibr. Day 0: fibroblasts before the hSTEMCCA infection. Fibr. Day 7: fibroblasts after 7 days of infection. Note the switch between the exogenous and the endogenous OCT4 during the reprogramming. (**C**) RT-PCR analysis of the indicated lineage-specific markers in iPSCs differentiated in vitro as floating embryoid bodies for 11 days. NESTIN, NCAM and NR2F2 are ectoderm markers; BRACHYURY and MIXL1 are mesendodermal markers; RUNX1 is a mesoderm marker; GATA4 is expressed in mesoderm and endoderm lineages; AFP and SOX17 are endoderm markers. GAPDH was used as a positive amplification control. (**D**) Sequencing of FUS and TDP-43 cDNA in ALS iPSC lines. The WT and mutant sequences are indicated below each panel. Arrows indicate the mutated base. Note a double peak for ALS I-FUS$^{R514S/WT}$ and ALS II-FUS$^{R521C/WT}$ (heterozygous mutants) and a single peak for ALS III-TDP43$^{A382T/A382T}$ (homozygous mutant). (**E**,**F**) WT I iPSCs do not contain mutations in FUS or TDP-43 genes. The entire coding sequence of FUS and TDP-43 has been sequenced from cDNA obtained from RNA of iPSCs WT I. Panels show the analysis of the sequences with the BLAT tool of the UCSC genome browser (UCSC Genome Browser: Kent WJ, Sugnet CW, Furey TS, Roskin KM, Pringle TH, Zahler AM, Haussler D. The human genome browser at UCSC. Genome Res. 2002 Jun;12(6):996-1006) (BLAT: Kent WJ. BLAT - the BLAST-like alignment tool. Genome Res. 2002 Apr;12(4):656-64.). BLAT analysis showed 100% identity between sequenced FUS and TDP-43 and the reference genome.

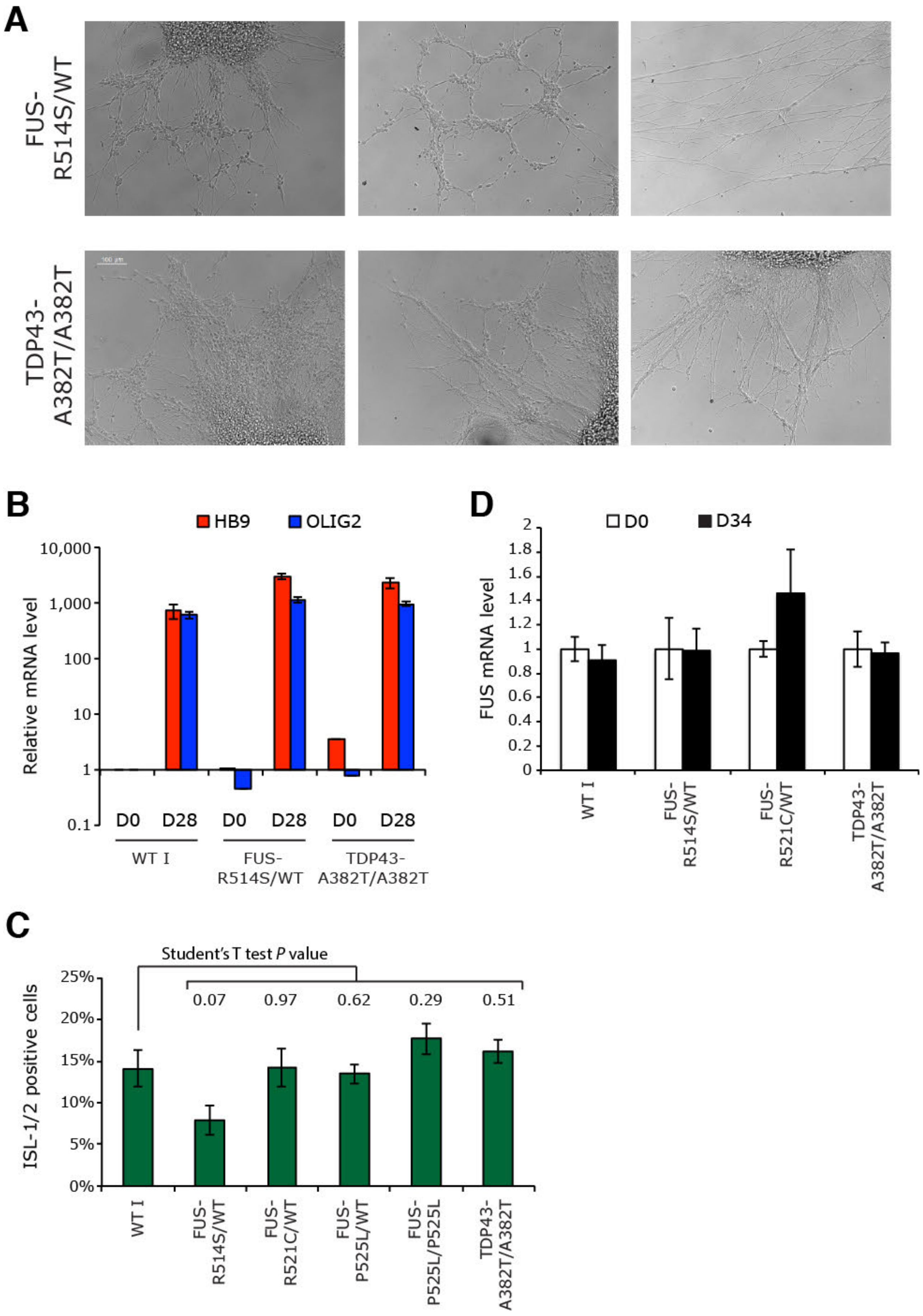
A
FUS-R514S/WT
TDP43-A382T/A382T
B
HB9
OLIG2
Relative mRNA level
10,000
1,000
100
10
1
0.1
D0
D28
WT I
FUS-R514S/WT
TDP43-A382T/A382T
C
Student's T test P value
ISL-1/2 positive cells
25%
20%
15%
10%
5%
0%
0.07
0.97
0.62
0.29
0.51
WT I
FUS-R514S/WT
FUS-R521C/WT
FUS-P525L/WT
FUS-P525L/P525L
TDP43-A382T/A382T
D
D0
D34
FUS mRNA level
2
1.8
1.6
1.4
1.2
1
0.8
0.6
0.4
0.2
0
WT I
FUS-R514S/WT
FUS-R521C/WT
TDP43-A382T/A382T

Figure S2 – Differentiation of iPSCs to ventral spinal cord derivatives.

(**A**) Representative images of neuronal cells observed in differentiating cultures at day 34. Scale bar for all panels: 100µm. (**B**) Real-time qRT-PCR analysis of the expression of the motoneuron precursor markers HB9 (red bars) and OLIG2 (blue bars) in undifferentiated (D0) and in differentiating (D28) iPSCs. Note the log scale. (**C**) Percentage of ISL-1/2 MN marker positive cells detected by immunostaining analysis in differentiated iPSCs (34 days). To count ISL-1/2 positive cells, we used the Count Nuclei Apps of the MetaMorph software, running the segmentation on the DAPI channel to estimate the total number of cells and on the ISL-1/2 channel to estimate the total number of MNs. We counted 1000-6000 cells per line. Statistical analysis (Student's T test) showed no significant difference in the differentiation ability of mutant iPSCs compared to the WT I line as all *P* values, indicated in the histogram, are above 0.05. (**D**) FUS mRNA levels, analyzed by real-time qRT-PCR, did not change during MN differentiation in control and patients' cell lines.

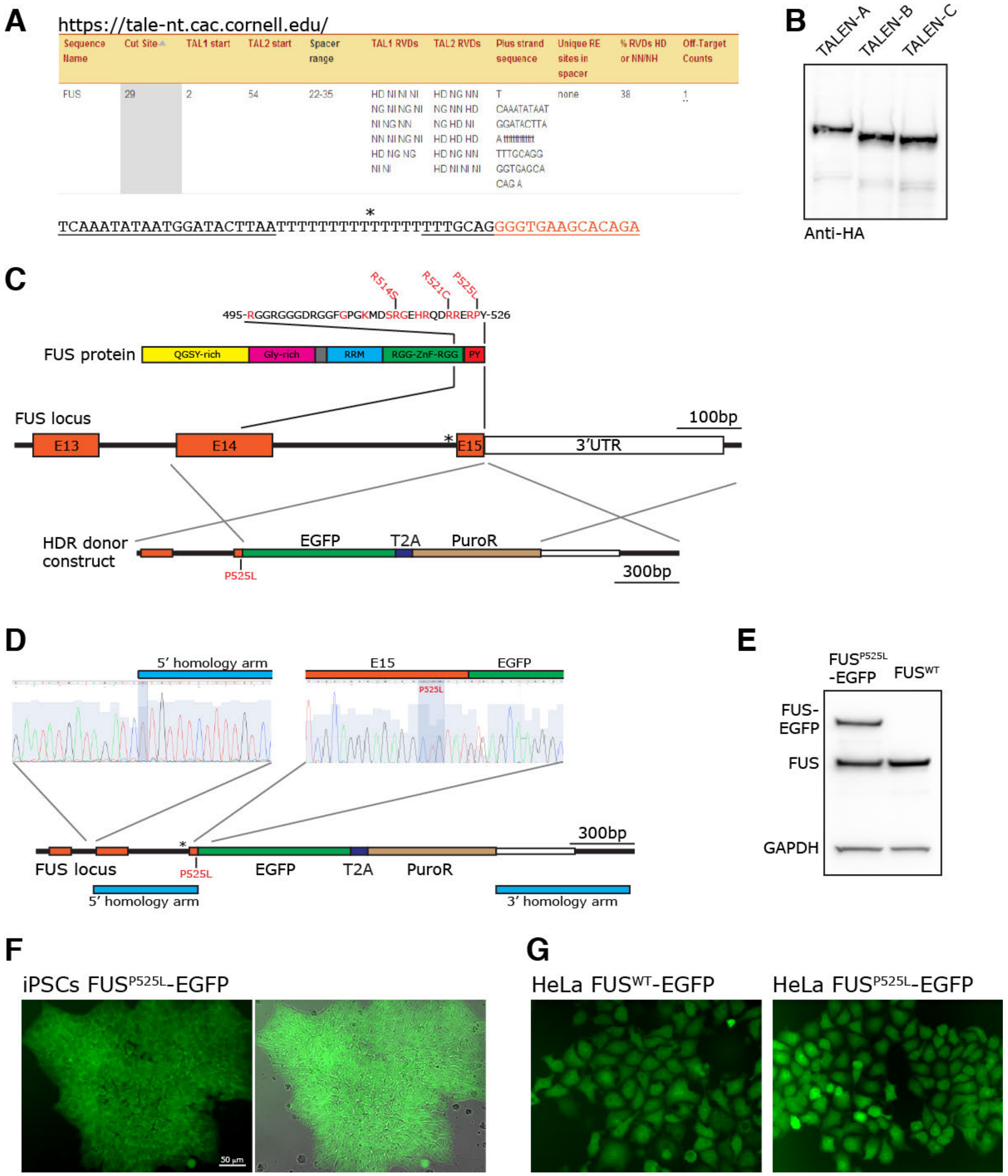

Figure S3 – TALEN-based mutagenesis of the FUS locus in iPSCs.

(**A**) The TALE-TN software (available at https://tale-nt.cac.cornell.edu/; ref. Doyle et al. TAL Effector-Nucleotide Targeter (TALE-NT) 2.0: tools for TAL effector design and target prediction. Nucleic Acids Res. 2012 doi: 10.1093/nar/gks608) provided the sequence of a TALEN pair (TLENS B-C, see below) specific for the region of interest in the FUS locus. The "Off-Target Counts" column on the right indicates that in the human genome there is only one

sequence that can be recognized by this TALEN pair, which corresponds to the target sequence on FUS. The sequence targeted by the TALEN pair B-C used in this study is specified below (black indicates the intron and orange the exon 15). Sequences bound by each TALEN are underlined. The asterisk indicates the predicted site of cleavage by TALENs. (**B**) Three TALENs have been generated and their size checked by western blot with an anti-HA specific antibody after transfection in HeLa cells. TALENs pair that can target the FUS locus are A-C and B-C. The assembling of the repeat-variable diresidue (RVD) of a TALEN can often lead to an aberrant protein containing more or less repeats than expected, often difficult to discern by sequencing. In this case, the TALEN-A showed higher molecular weight than expected. For this reason we used the B-C pair (FUS C-term TALENs) for subsequent mutagenesis experiments. (**C**) The activity of the FUS C-term TALENs was assayed by stimulation of Homology Directed Recombination (HDR) of a donor construct containing a reporter/selection cassette flanked by homology arms carrying the P525L mutation. In this construct, the EGFP sequence is fused to the puromycin resistance sequence (PuroR) through a T2A self-cleavage peptide. The EGFP CDS is devoid of the start codon and in frame with the last FUS exon. In this way, only a precise HDR event stimulated by TALENs could lead to the production of a chimeric FUS-EGFP protein and PuroR. Upon cotransfection with the FUS C-term TALENs, this construct conferred resistance to puromycin. The panel shows on the top the different domains of the FUS protein. Amino acid residues 495-526, including the C-term nuclear localization signal and encoded by exon 15 and part of exon 14, are specified. Red color denotes residues frequently mutated in ALS. In particular, the mutations analyzed in the present study are indicated. In the middle part of the scheme, the structure of the FUS locus is shown in scale. Exons 13-15 are depicted as orange boxes, introns as black lines and the 3'UTR as a white box. The asterisk indicates the site cleaved by FUS C-term TALENs. On the bottom, the donor construct used for homologous recombination (HDR). Grey lines represent recombinations between the FUS locus and the homology arms. EGFP: enhanced Green Fluorescent Protein; T2A: self-cleavage peptide; PuroR: puromycin resistance gene. Note the different scale. (**D**) Genomic DNA sequencing of FUS^{P525L}-EGFP iPSCs confirming the in-frame fusion of the EGFP to FUS exon 15 and the presence of the specific mutation of interest, i.e. a CCG to CTG shift in codon 525 leading to Prolin to Leucin substitution in the FUS C-terminal domain. The boundary of the 5' homology arm, the P525L mutation and the boundary between FUS exon 15 (devoid of the stop codon) and EGFP (devoid of the start codon) are showed. (**E**) Western blot analysis of the endogenous FUS protein in $FUSP^{525L}$-EGFP and parental FUS^{WT} iPSCs, showing a higher molecular weight band in transfected iPSCs,

corresponding to the expected FUS^{P525L}-EGFP chimeric protein. Untagged FUS and FUS-EGFP proteins are recognized by an anti-FUS antibody. An anti-GAPDH antibody is used for loading control. (**F**) Live cell imaging showing a green fluorescence signal in FUS^{P525L}-EGFP iPSCs. Left: EGFP signal. Right: merge between EGFP and DIC. Scale bar for both panels: 50μm. (**G**) As shown in panel (E) the FUS^{P525L}-EGFP protein was localized both in the nucleus and in the cytoplasm. However, the loss of FUS nuclear localization was conferred by the EGFP tag also in absence of the P525L mutation, suggesting that tagging the FUS protein at the C-terminal is sufficient to disrupt the function of the PY NLS. The panel shows HeLa cells co-transfected with FUS C-term TALENs and either the HDR donor construct depicted in panel (C) or a similar construct containing a WT 525 codon. Live cell imaging of FUS-EGFP localization in these cells after puromycin selection shows the diffuse localization of the protein in both conditions.

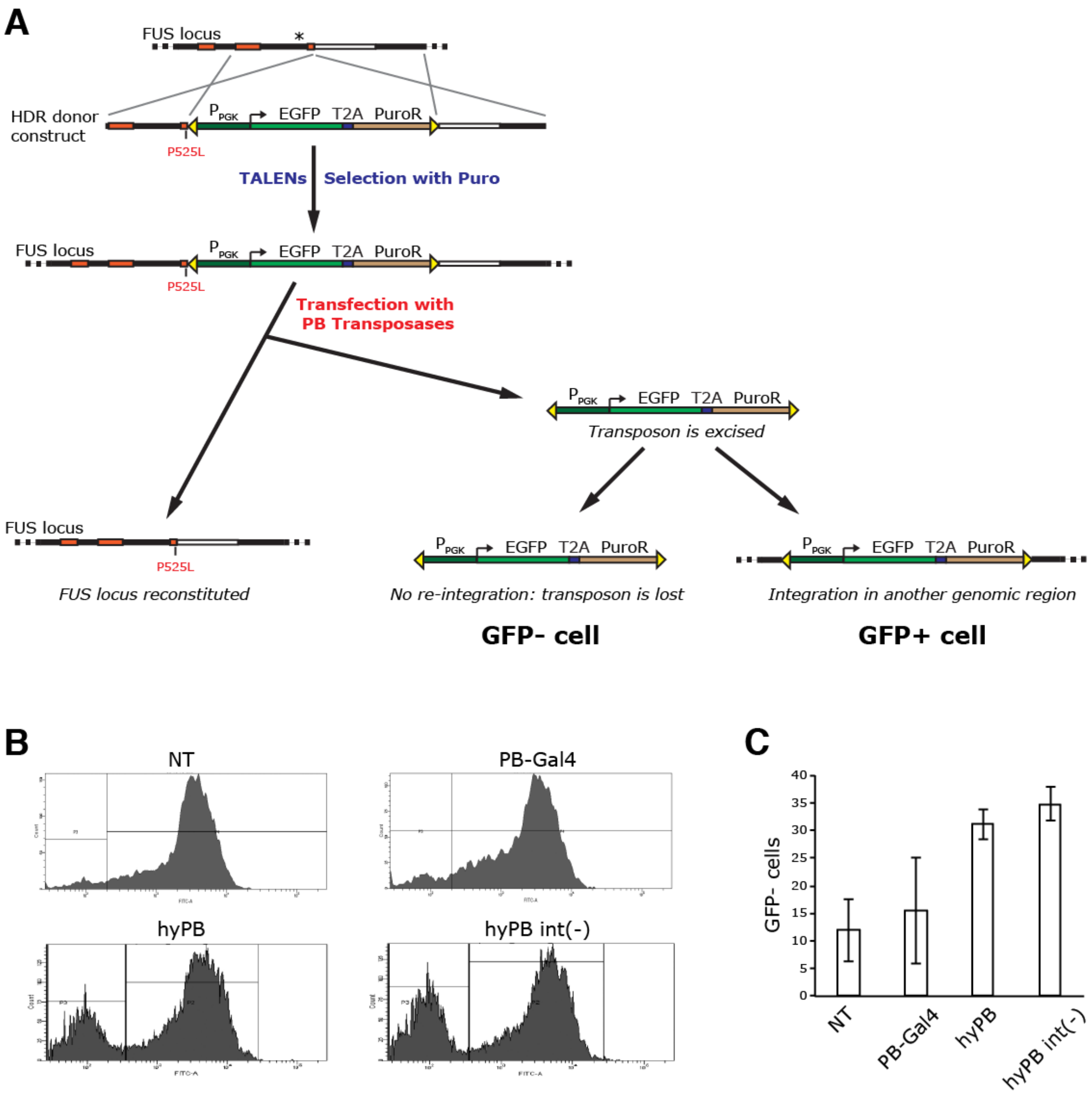

Figure S4 – Optimization of the removal of the selection cassette in TALEN-targeted iPSCs.

(**A**) Schematic representation of the combined TALEN/piggyBac strategy to optimize the removal of an EGFP-PuroR reporter/selection cassette from the P525L FUS mutant. After transfection with a piggyBac (PB) transposase, the sequence flanked by the PB terminal repeats (yellow triangles) can be integrated elsewhere in the genome (in this case the cell will maintain the green fluorescence) or lost (in this case the cell will lose the green fluorescence). (**B**) Representative FACS analyses of HeLa cells modified as in (**A**) with TALENs and transfected with different variants of the PB transposase, or left untreated (NT). PB-Gal4: PB transposase fused to the Gal4 DNA binding domain and cotransfected with a plasmid containing multiple UAS. This transposase should preferentially integrate the transposon in

the UAS plasmid (Maragathavally et al., FASEB J 2006; Lacoste et al., 2009). hyPB: "hyperactive" mutant PB transposase (Yusa et al., 2013). hyPB int(-): hyPB further modified with mutations that abolish its integration activity (Li et al., 2013). Note peaks on the left (P3 quadrant), indicating an increased fraction of GFP-negative cells in hyPB and hyPB int(-) samples. (**C**) Histogram averaging different experiments (3 for NT and PB-Gal4; 2 for hyPB and hyPB int(-)) analyzed by FACS as in (B).

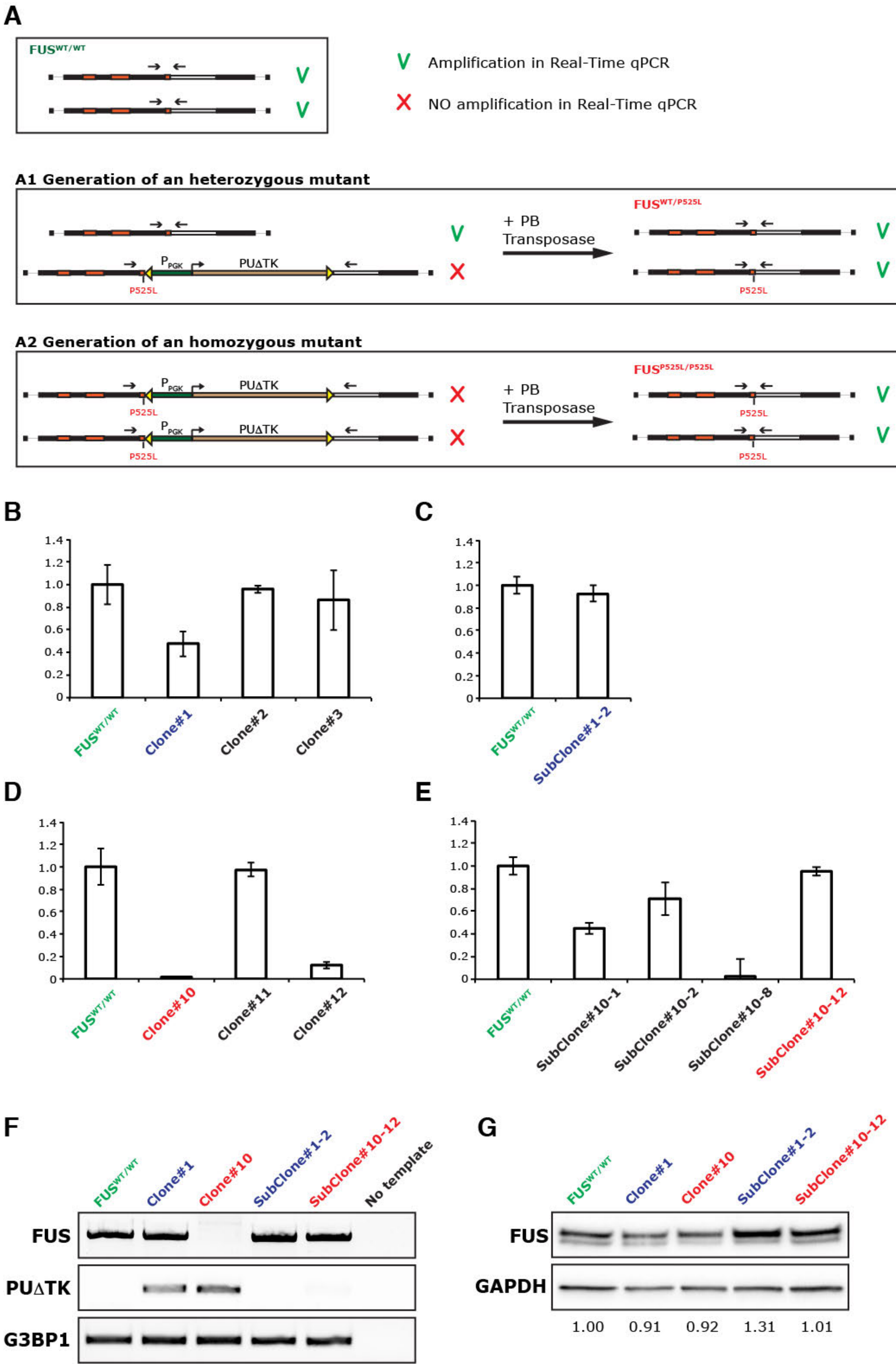
A
FUS WT/WT
Amplification in Real-Time qPCR
NO amplification in Real-Time qPCR
A1 Generation of an heterozygous mutant
FUS WT/P525L
+ PB Transposase
PPGK
PUΔTK
P525L
A2 Generation of an homozygous mutant
FUS P525L/P525L
B
FUS WT/WT
Clone#1
Clone#2
Clone#3
C
SubClone#1-2
D
Clone#10
Clone#11
Clone#12
E
SubClone#10-1
SubClone#10-2
SubClone#10-8
SubClone#10-12
F
No template
FUS
PUΔTK
G3BP1
G
GAPDH
1.00
0.91
0.92
1.31
1.01

Figure S5 – Analysis of FUS^{P525L} iPSC clones

(**A**) Schematic representation of the real-time qPCR analysis on the genomic DNA of WT and mutant iPSCs, transfected with FUS C-term TALENs and the HDR donor construct depicted in Figure 4. Primers used for real-time qPCR are shown as arrows. These primers will amplify the template only in the absence of the selection cassette. The genome of WT untransfected iPSCs will give amplification from both FUS loci (top panel). If TALEN-directed HDR resulted in the integration of the selection cassette (and the mutation) in one locus, only the other locus will be amplified (A1 panel). Conversely, amplification will be completely impaired if both loci were targeted (A2 panel). After PB transposase-directed removal of the selection cassette, in both cases the level of amplification will be reverted to that of the WT cells. Therefore, in a relative quantification, we expect as 1 the PCR product of two loci unmodified, as 0.5 the PCR product of one targeted/one unmodified locus, and as 0 the case of both loci targeted. Any intermediate value would be indicative of the presence of a non-clonal population of cells. (**B**,**D**) iPSCs transfected with the FUS C-term TALENs and the HDR donor construct were selected with puromycin and expanded as individual clones. The histograms refer to two different experiments and show the representative analysis, as in (A), of 3 clones and the parental line FUS$^{WT/WT}$. Note that clone#1 (in panel B) and clone#10 (in panel D) show, respectively, about 50% and 0% amplification compared to the parental cells, suggesting a heterozygous situation in clone#1 and a homozygous situation in clone #10 as depicted in panels A1 and A2, left. Clones #2, #3 and #11 are close to the parental line: in these cases we hypothesize random integration of the selection cassette elsewhere in the genome. Clone#12 levels are intermediate, suggesting a mixed nature of this population. (**C**,**E**) iPSCs clones #1 and #10 were transfected with the hyPB int(-) transposase, selected with ganciclovir and expanded as individual subclones. The histogram shows the representative analysis of 2 and 4 subclones and the parental line FUS$^{WT/WT}$. Note that the subclones #1-2 and #10-12 are reverted to an amplification level close to 1, as depicted in panel A1 and A2, right. Figure 3B shows the results of genomic sequencing of subclones #1-2 (heterozygous) and #10-12 (homozygous). (**F**) End-point PCR analysis on the genomic DNA of WT and mutant iPSCs with primers depicted in panel (A) (FUS), or with primers annealing in the selection cassette (PUΔTK), or with control primers annealing in an unrelated locus on the genome (G3BP1). Amplification of the selection cassette, clearly detectable in clones #1 and #10, was undetectable in subclones #1-2 and #10-12 after PB transposase transfection and ganciclovir selection. (**G**) Western blot analysis of FUS protein levels in parental WT I iPSCs and in clones #1 and #10 and subclones #1-2 and #10-12. Anti-FUS/TLS (sc-47711, Santa

Cruz) and, as loading control, anti-GAPDH (sc-32233, Santa Cruz) antibodies were used. Densitometric quantification of FUS protein, relative to WT I, is shown below (average from a technical triplicate).

A

```
CLUSTAL O(1.2.1) multiple sequence alignment


FUS-WT                -----------------------------------------GGGGAATGGGAATATGATA     19
FUS-P525L/WT          ---------------------GGGGAGGCTCGGGGAACATAGGGGAATGGGAATATGATA     39
FUS-P525L/P525L       CGGGGAGGCTCGGGGAACATAGGGGAGGCTCGGGGAACATAGGGGAATGGGAATATGATA     60
                                                               *******************

FUS-WT                GATCTTGTTTCTTTTGTCCTAGGGGGTAACTACGGGGATGATCGTCGTGGTGGCAGAGGA     79
FUS-P525L/WT          GATCTTGTTTCTTTTGTCCTAGGGGGTAACTACGGGGATGATCGTCGTGGTGGCAGAGGA     99
FUS-P525L/P525L       GATCTTGTTTCTTTTGTCCTAGGGGGTAACTACGGGGATGATCGTCGTGGTGGCAGAGGA     120
                      ************************************************************

FUS-WT                GGCTATGATCGAGGCGGCTACCGGGGCCGCGGCGGGGACCGTGGAGGCTTCCGAGGGGGC     139
FUS-P525L/WT          GGCTATGATCGAGGCGGCTACCGGGGCCGCGGCGGGGACCGTGGAGGCTTCCGAGGGGGC     159
FUS-P525L/P525L       GGCTATGATCGAGGCGGCTACCGGGGCCGCGGCGGGGACCGTGGAGGCTTCCGAGGGGGC     180
                      ************************************************************

FUS-WT                CGGGGTGGTGGGGACAGAGGTGGCTTTGGCCCTGGCAAGATGGATTCCAGGTAAGACTTT     199
FUS-P525L/WT          CGGGGTGGTGGGGACAGAGGTGGCTTTGGCCCTGGCAAGATGGATTCCAGGTAAGACTTT     219
FUS-P525L/P525L       CGGGGTGGTGGGGACAGAGGTGGCTTTGGCCCTGGCAAGATGGATTCCAGGTAAGACTTT     240
                      ************************************************************

FUS-WT                AAATCAGAATAAAAAAGTAGAGCAGTTGAACAGAGGCCATAGGATAACAGGGTTTTGTTG     259
FUS-P525L/WT          AAATCAGAATAAAAAAGTAGAGCAGTTGAACAGAGGCCATAGGATAACAGGGTTTTGTTG     279
FUS-P525L/P525L       AAATCAGAATAAAAAAGTAGAGCAGTTGAACAGAGGCCATAGGATAACAGGGTTTTGTTG     300
                      ************************************************************

FUS-WT                AGAAAGTGGTTTCATTTTGAGGGCTAGGTGGAAAGACCTGAGGTTGTAACCAGTAGTGGA     319
FUS-P525L/WT          AGAAAGTGGTTTCATTTTGAGGGCTAGGTGGAAAGACCTGAGGTTGTAACCAGTAGTGGA     339
FUS-P525L/P525L       AGAAAGTGGTTTCATTTTGAGGGCTAGGTGGAAAGACCTGAGGTTGTAACCAGTAGTGGA     360
                      ************************************************************

FUS-WT                GAGGGAAGGAAAATTAACTCAGGGGGAGTGAATCTGTAGACCCACTTGAGATAAGATACT     379
FUS-P525L/WT          GAGGGAAGGAAAATTAACTCAGGGGGAGTGAATCTGTAGACCCACTTGAGATAAGATACT     399
FUS-P525L/P525L       GAGGGAAGGAAAATTAACTCAGGGGGAGTGAATCTGTAGACCCACTTGAGATAAGATACT     420
                      ************************************************************

FUS-WT                CGCTGGGTTAGGTAGGAGGGGCAGATAGGATATCTAGGCTTGGAGAGGCTGGTAACTCAA     439
FUS-P525L/WT          CGCTGGGTTAGGTAGGAGGGGCAGATAGGATATCTAGGCTTGGAGAGGCTGGTAACTCAA     459
FUS-P525L/P525L       CGCTGGGTTAGGTAGGAGGGGCAGATAGGATATCTAGGCTTGGAGAGGCTGGTAACTCAA     480
                      ************************************************************

FUS-WT                ATATAATGGATACTTAATTTTTTTTTTTTTTTTTGCAGGGGTGAGCACAGACAGGATCGC     499
FUS-P525L/WT          ATATAATGGATACTTAATTTTTTTTTTTTTTTTTGCAGGGGTGAGCACAGACAGGATCGC     519
FUS-P525L/P525L       ATATAATGGATACTTAATTTTTTTTTTTTTTTTTGCAGGGGTGAGCACAGACAGGATCGC     540
                      ************************************************************

FUS-WT                AGGGAGAGGCCGTATTAATTAGCCTGGCTCCCCAGGTTCTGGAACAGCTTTTTGTCCTGT     559
FUS-P525L/WT          AGGGAGAGGCNGTATTAATTAGCCTGGCTCCCCAGGTTCTGGAACAGCTTTTTGTCCTGT     579
FUS-P525L/P525L       AGGGAGAGGCTGTATTAATTAGCCTGGCTCCCCAGGTTCTGGAACAGCTTTTTGTCCTGT     600
                      ********** *************************************************

FUS-WT                ACCCAGTGTTACCCTCGTTATTTTGTAACCTTCCAATTCCTGATCACCCAAGGGTTTTTT     619
FUS-P525L/WT          ACCCAGTGTTACCCTCGTTATTTTGTAACCTTCCAATTCCTGATCACCCAAGGGTTTTTT     639
FUS-P525L/P525L       ACCCAGTGTTACCCTCGTTATTTTGTAACCTTCCAATTCCTGATCACCCAAGGGTTTTTT     660
                      ************************************************************

FUS-WT                TGTGTCGGACTATGTAATTGTAACTATACCTCTGGTTCCCATTAAAAGTGACCATTTTAG     679
FUS-P525L/WT          TGTGTCGGACTATGTAATTGTAACTATACCTCTGGTTCCCATTAAAAGTGACCATTTTAG     699
FUS-P525L/P525L       TGTGTCGGACTATGTAATTGTAACTATACCTCTGGTTCCCATTAAAAGTGACCATTTTAG     720
                      ************************************************************

FUS-WT                TTAAATTTTGTTCCTCTTCCCCCTTTTCACTTTCCTGGAAGATCGATGTCCCGATCAGGA     739
FUS-P525L/WT          TTAAATTTTGTTCCTCTTCCCCCTTTTCACTTTCCTGGAAGATCGATGTCCCGATCAGGA     759
FUS-P525L/P525L       TTAAATTTTGTTCCTCTTCCCCCTTTTCACTTTCCTGGAAGATCGATGTCCCGATCAGGA     780
                      ************************************************************

FUS-WT                AGGTAGAGAGTTTTCCTGTTCAGATTACCCTGCCCAGCAGGAACTGGAATACAGTGTTCG     799
FUS-P525L/WT          AGGTAGAGAGTTTTCCTGTTCAGATTACCCTGCCCAGCAGGAACTGGAATACAGTGTTCG     819
FUS-P525L/P525L       AGGTAGAGAGTTTTCCTGTTCAGATTACCCTGCCCAGCAGGAACTGGAATACAGTGTTCG     840
                      ************************************************************

FUS-WT                GGGAGAAGGCCAAATGATATCCTTGAGAGCAGAGATTAAACTTTTCTGTCATGGGGAAAG     859
FUS-P525L/WT          GGGAGAAGGCCAAATGATATCCTTGAGAGCAGAGATTAAACTTTTCTGTCATGGGGAAAG     879
FUS-P525L/P525L       GGGAGAAGGCCAAATGATATCCTTGAGAGCAGAGATTAAACTTTTCTGTCATGGGGAAAG     900
                      ************************************************************

FUS-WT                TTGGTGTATAAATGAGAAATGAAGAACATGGGATGTCATGAGTGTTGGCCTAAATTTGCC     919
FUS-P525L/WT          TTGGTGTATAAATGAGAAATGAAGAACATGGGATGTCATGAGTGTTGGCCTAAATTTGCC     939
FUS-P525L/P525L       TTGGTGTATAAATGAGAAATGAAGAACATGGGATGTCATGAGTGTTGGCCTAAATTTGCC     960
                      ************************************************************

FUS-WT                CAGCTATGGGGAATTTTTCCTTTACCACATTTATTTGCATACTGGTCTTAGTTTATTTGC     979
FUS-P525L/WT          CAGCTATGGGGAATTTTTCCTTTACCACATTTATTTGCATACTGGTCTTAGTTTATTTGC     999
FUS-P525L/P525L       CAGCTATGGGGAATTTTTCCTTTACCACATTTATTTGCATACTGGTCTTAGTTTATTTGC     1020
                      ************************************************************

FUS-WT                AGCAGTTTATCCCTTTTTAAGAACTCTTTGATCTTTTGGCCCTTTTAATGGTGAGGCTCA     1039
FUS-P525L/WT          AGCAGTTTATCCCTTTTTAAGAACTCTTTGATCTTTTGGCCCTTTTAATGGTGAGGCTCA     1059
FUS-P525L/P525L       AGCAGTTTATCCCTTTTTAAGAACTCTTTGATCTTTTGGCCCTTTTAATGGTGAGGCTCA     1080
                      ************************************************************

FUS-WT                AACAAACTACATTTAAATGGGGCAGTATTCAGATTTGACCATGGTGGAGAGCGCTTAGCC     1099
FUS-P525L/WT          AACAAACTACATTTAAATGGGGCAGTATTCAGATTTGACCATGGTGGAGAGCGCTTAGCC     1119
FUS-P525L/P525L       AACAAACTACATTTAAATGGGGCAGTATTCAGATTTGACCATGGTGGAGAGCGCTTAGCC     1140
                      ************************************************************

FUS-WT                ACTCTGGGTCTTTCACAGGAAGGAGAGTAACTGAGTGCTGCAGGAGTTTGGGAGGGAGTC     1159
FUS-P525L/WT          ACTCTGGGTCTTTCACAGGAAG--------------------------------------     1141
FUS-P525L/P525L       ACTCTGGGTCTTTCACAGGAAG--------------------------------------     1162
```

B

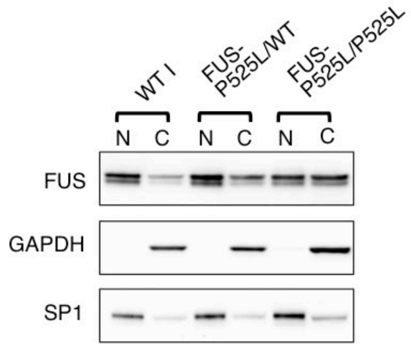

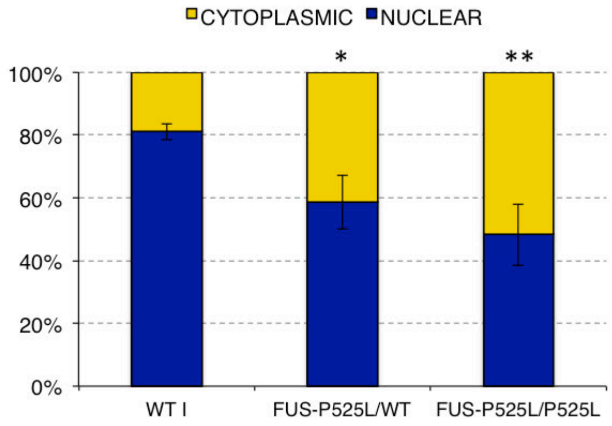

Figure S6 – Sequencing of FUS^{P525L} iPSC clones and nuclear/cytoplasmic distribution of FUS protein

(**A**) A fragment corresponding to about 1 kb of genomic DNA around the mutation site was PCR amplified from heterozygous (FUS-P525L/WT) or homozygous (FUS-P525L/P525L) iPSC lines and sequenced. The figure shows the alignment of the sequences with the reference genome performed by the ClustalO software (http://www.ebi.ac.uk/Tools/msa/clustalo/). No mutation was found in either clone outside the targeted site (corresponding to nucleotide 509 in the FUS-WT sequence). (**B**) FUS protein distribution in nuclear/cytoplasmic compartments. Cell lysis and extraction of separate cytoplasmic and nuclear protein fractions from undifferentiated iPSCs were performed with the NE-PER kit (Thermo Scientific). Left panel: western blot was performed with anti-FUS/TLS (sc-47711, Santa Cruz) and, as fractionation controls, anti-SP1 (sc-59, Santa Cruz) and anti-GAPDH (sc-32233, Santa Cruz) antibodies for nuclear and cytoplasmic fractions, respectively. Right panel: densitometric analysis of western blots signals from four technical replicates. Statistically significant differences from WT I are indicated by asterisks (unpaired Student's t-test: *P<0.05, **P<0.01).

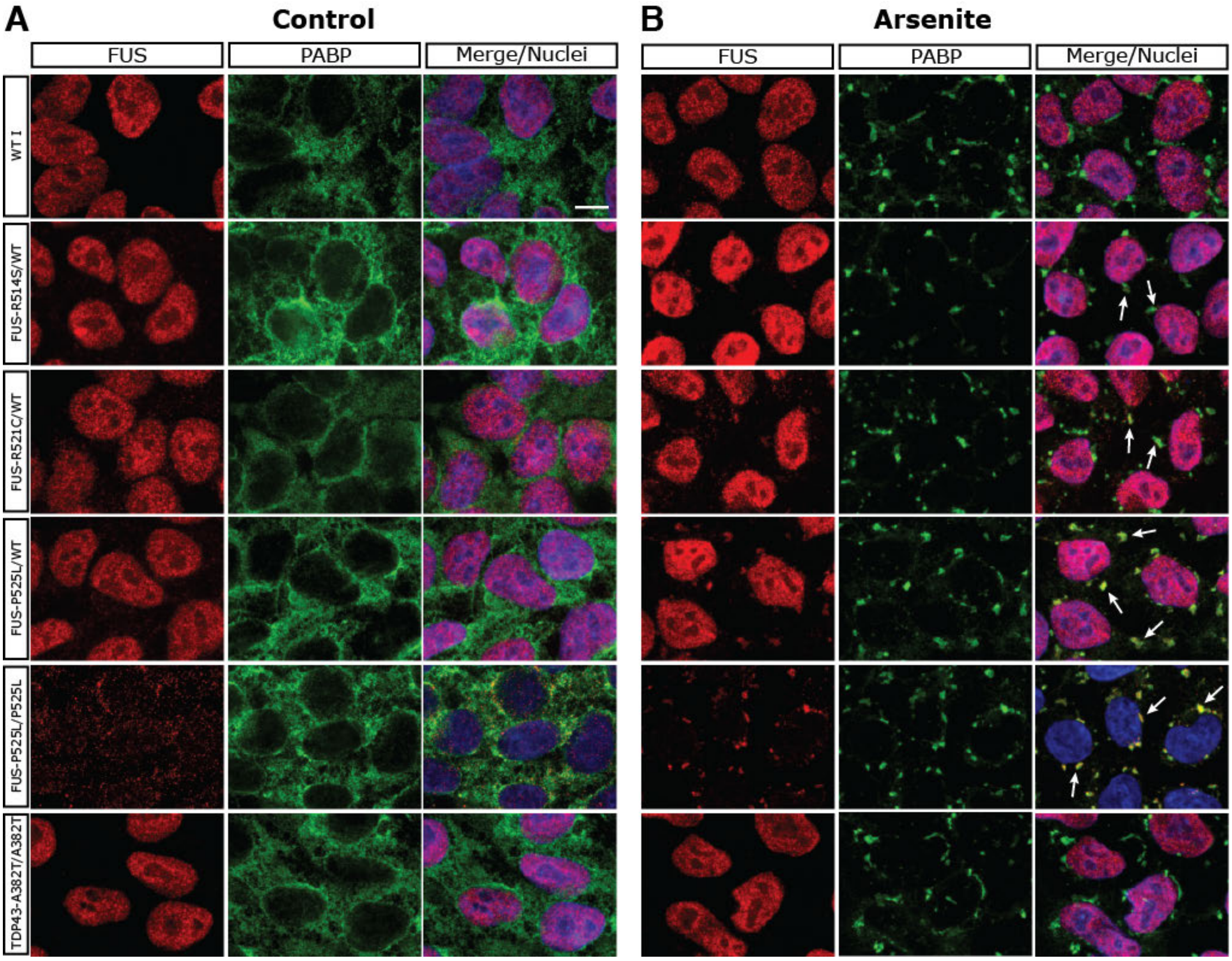

Figure S7 – Mutant FUS localized in PABP+ stress granules upon oxidative stress.

(**A-B**) Immunostaining of FUS (red) and the stress granule marker PABP (green) in undifferentiated iPSCs in control (untreated) conditions (**A**) or upon 0.5mM arsenite-induced oxidative stress for 60 minutes (**B**). Merge/Nuclei panels show the combined signals of FUS, PABP and DAPI. Scale bar for all panels: 10μm. Arrows indicate examples of co-localization of PABP and FUS signals in the cytoplasm.

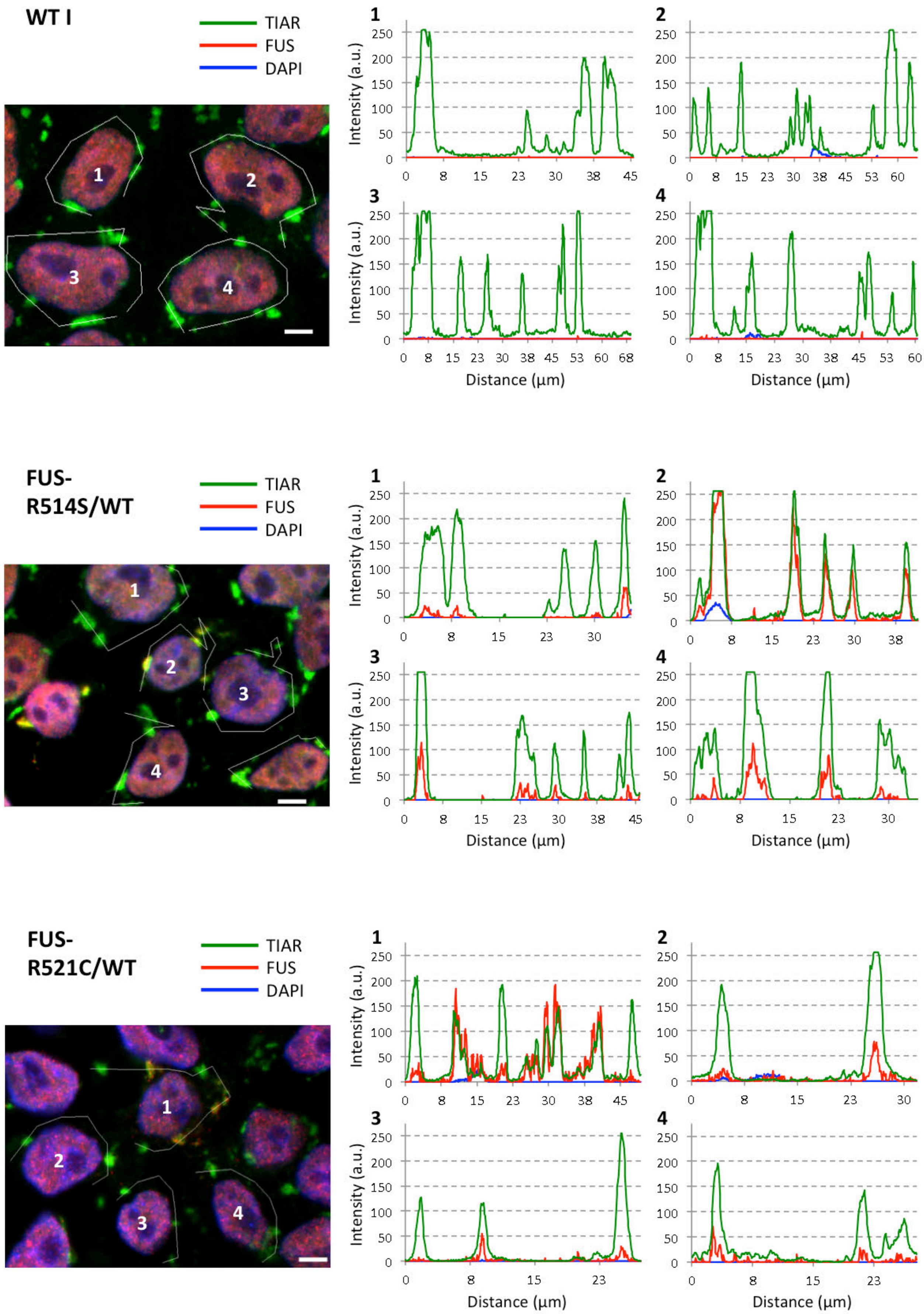
WT I
TIAR
FUS
DAPI
FUS-
R514S/WT
FUS-
R521C/WT
Intensity (a.u.)
Distance (µm)

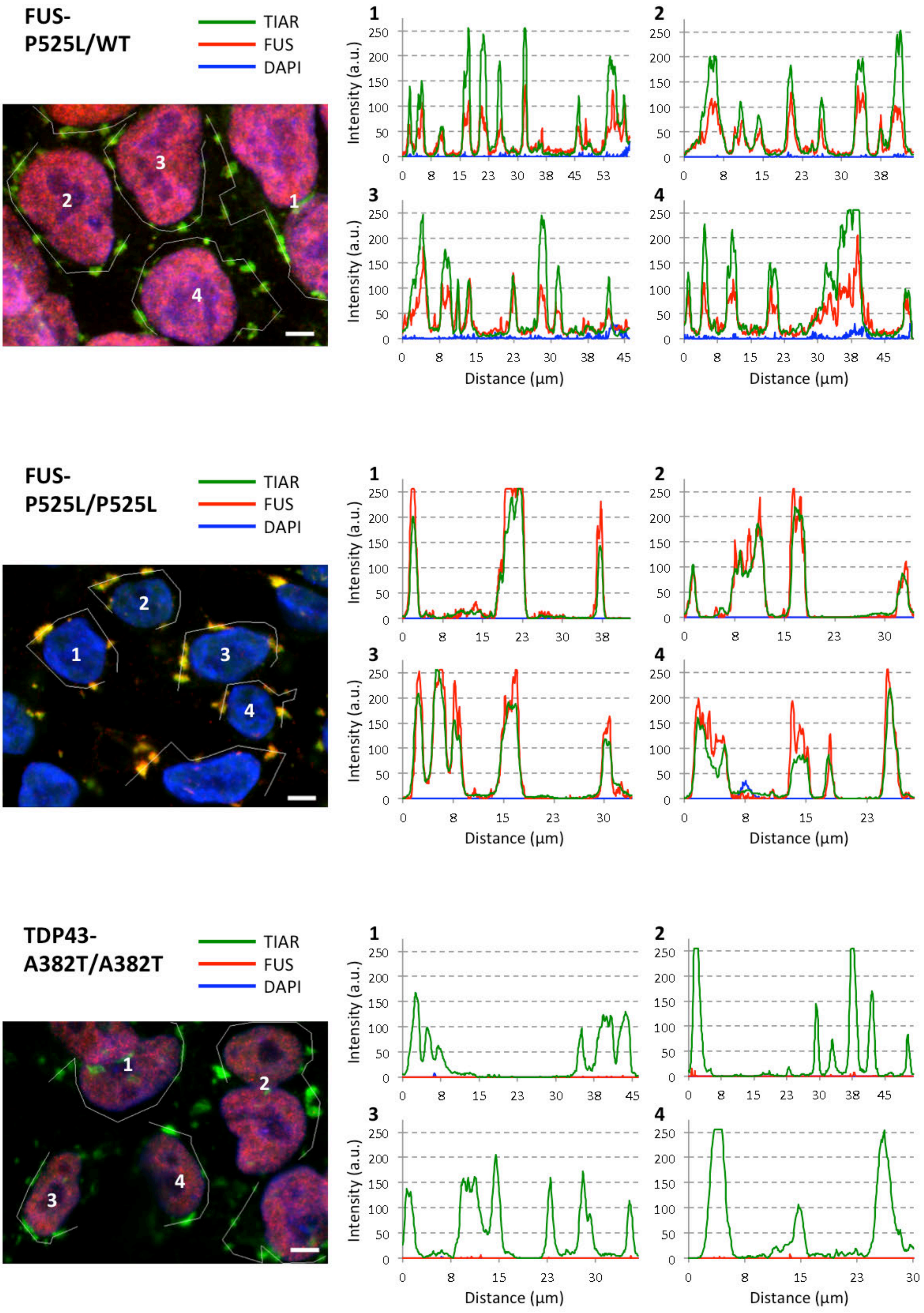
FUS-P525L/WT
TIAR
FUS
DAPI
1
2
3
4
Intensity (a.u.)
0 8 15 23 30 38 45 53
0 8 15 23 30 38
0 8 15 23 30 38 45
Distance (μm)
FUS-P525L/P525L
TIAR
FUS
DAPI
1
2
3
4
Intensity (a.u.)
0 8 15 23 30 38
0 8 15 23 30
0 8 15 23
Distance (μm)
TDP43-A382T/A382T
TIAR
FUS
DAPI
1
2
3
4
Intensity (a.u.)
0 8 15 23 30 38 45
0 8 15 23 30
Distance (μm)
0 50 100 150 200 250

Figure S8 – Linescan analysis of ARS-treated iPSCs

Co-localization of FUS and TIAR within SGs was analyzed by linescan (see details in the Methods section). In each IF panel shown on the left, a line crossing TIAR-positive SGs was drawn in cells numbered 1-4. On the right, the graphs show the corresponding signal intensities of FUS (red), TIAR (green) and DAPI (blue), in arbitrary units, along the line.

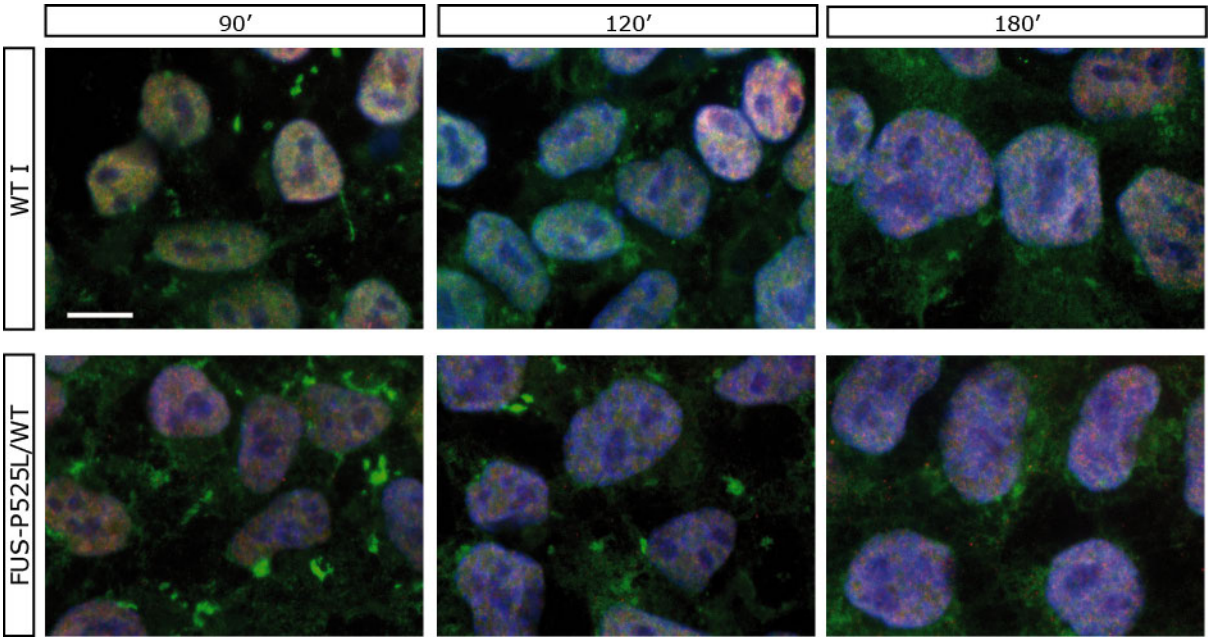

Figure S9 – Dissolution of SGs upon removal of stress

Immunostaining of FUS (red) and the stress granule marker TIAR (green) in undifferentiated iPSCs. Cells were cultured in presence of 0.5mM arsenite for 60 minutes, then washed and allowed to recover from stress in normal medium for the indicated time points (90'-120'-180'). Blue: DAPI. Scale bar for all panels is 10µm.

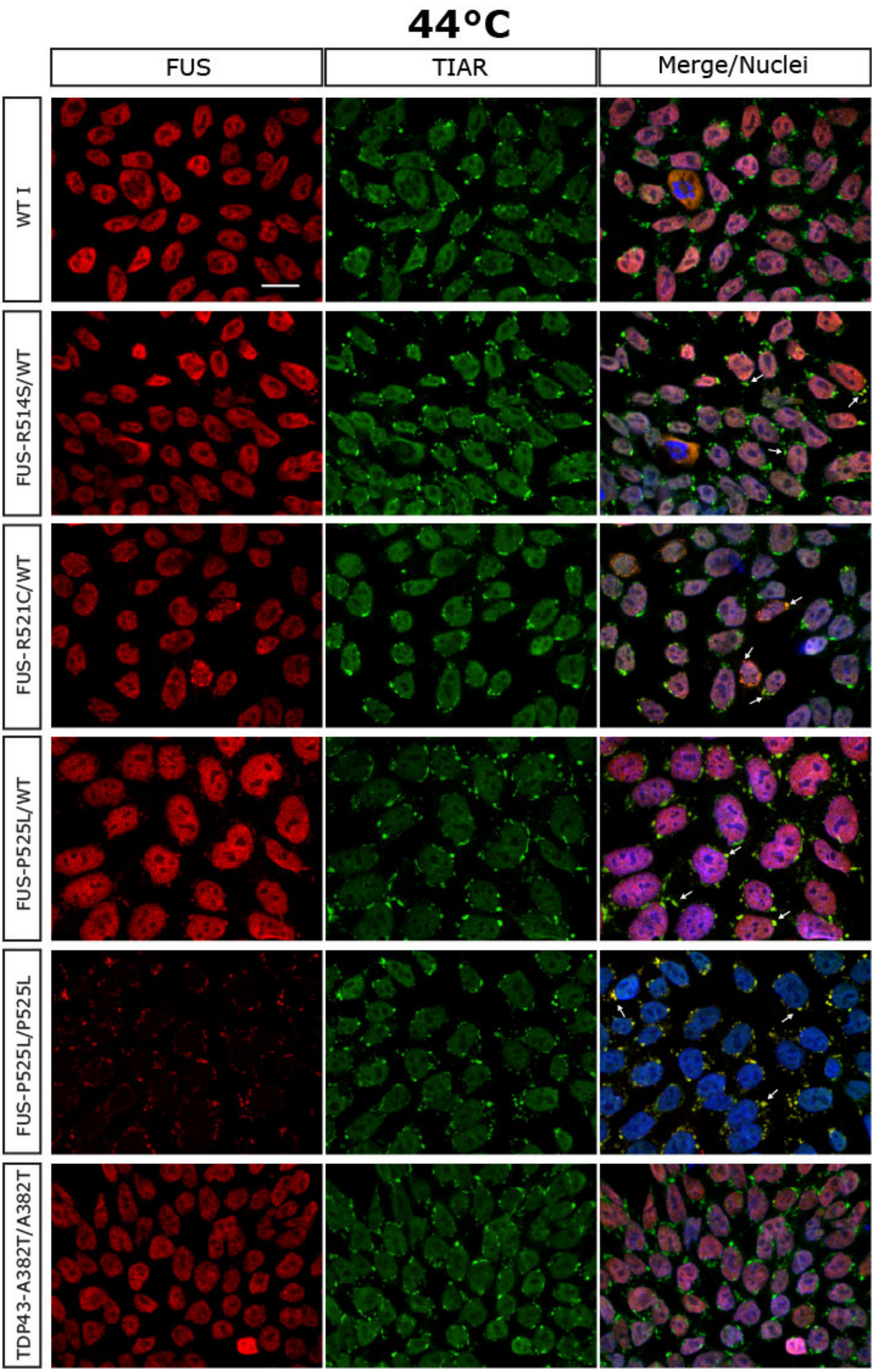
44°C
FUS
TIAR
Merge/Nuclei
WT I
FUS-R514S/WT
FUS-R521C/WT
FUS-P525L/WT
FUS-P525L/P525L
TDP43-A382T/A382T

Figure S10 – Mutant FUS localized in SGs during heat shock in undifferentiated iPSCs

Immunostaining of FUS (red) and the stress granule marker TIAR (green) in undifferentiated iPSCs cultured at 44°C for one hour. Blue: DAPI. Scale bar for all panels is 20μm.

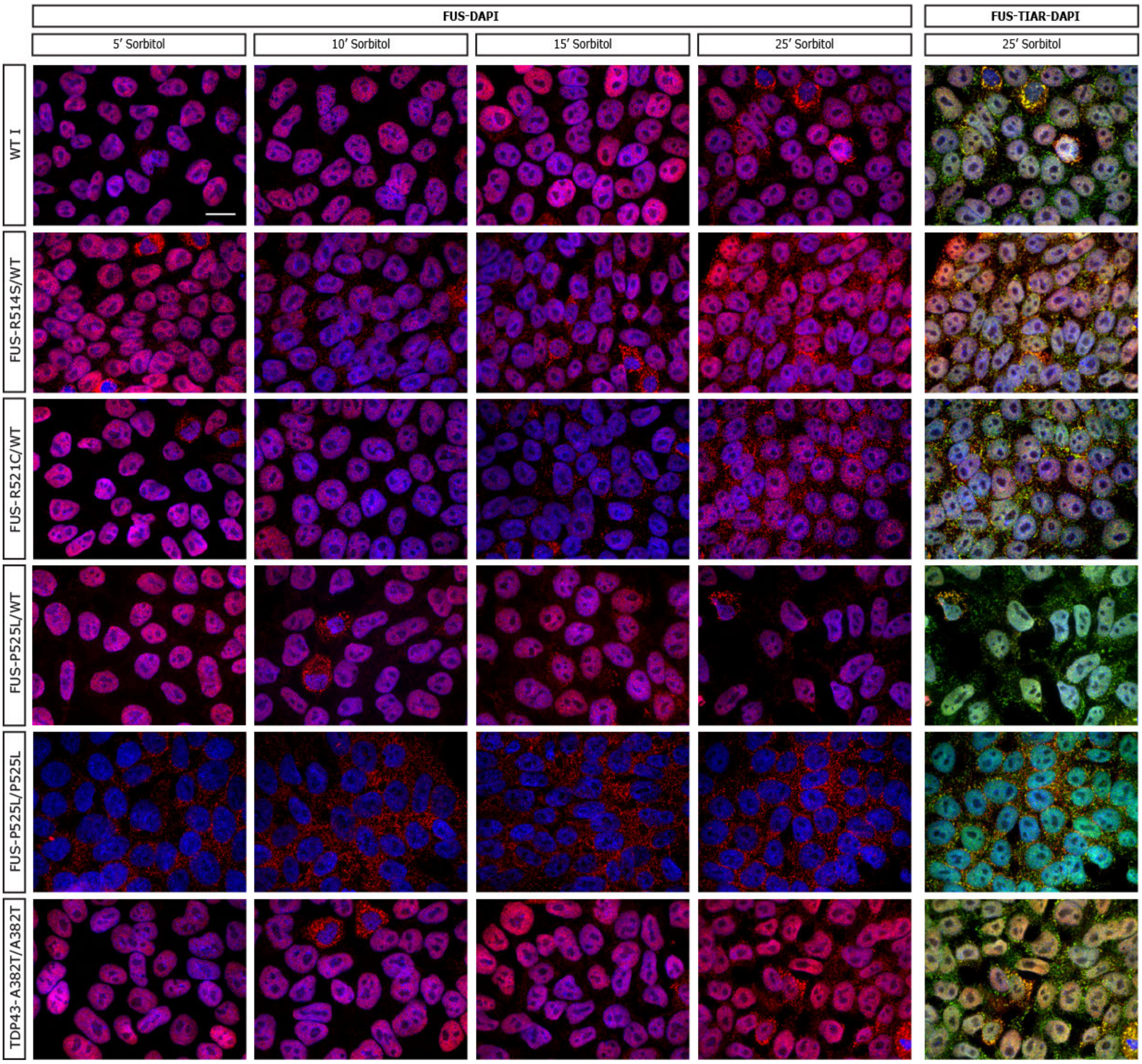

Figure S11 – Time course analysis of FUS localization during hyperosmolar stress

Immunostaining of FUS (red, all panels) and TIAR (panels on the right) in undifferentiated iPSCs exposed to 0.4M sorbitol for the indicated time points. Blue: DAPI (all panels). Scale bar for all panels is 20µm.

Note that cytoplasmic FUS partially co-localized with TIAR. Notably, compared to other kinds of stress, SGs are not completely formed after 25 minutes of hyperosmolar stress, as suggested by a fraction of TIAR signal still dispersed in the cytoplasm (compare TIAR panels in Figure 4, S10 and S11). As cytoplasmic FUS and TIAR do not co-localize outside SGs (Figure 4 and S12), the partial co-localization after 25 minutes of hyperosmolar stress may reflect an intermediate situation in which only a fraction of TIAR is engaged in SGs, and thus co-

localized with FUS, while the rest is still diffuse, and not co-localized with FUS. In this experiment we could not analyze further time points, as longer exposure to sorbitol caused massive cell death in undifferentiated iPSCs (data not shown). However, a clear increase in the amount of FUS in the cytoplasm in the mutants, compared to WT, could be detected in sorbitol at this time point. This evidence was also supported by the time-course analysis, in which mutant FUS^{R514S} and FUS^{R521C} proteins, that are almost exclusively nuclear in control conditions (Figure 3E), accumulate in the cytoplasm as early as 10-15 minutes after sorbitol exposure.

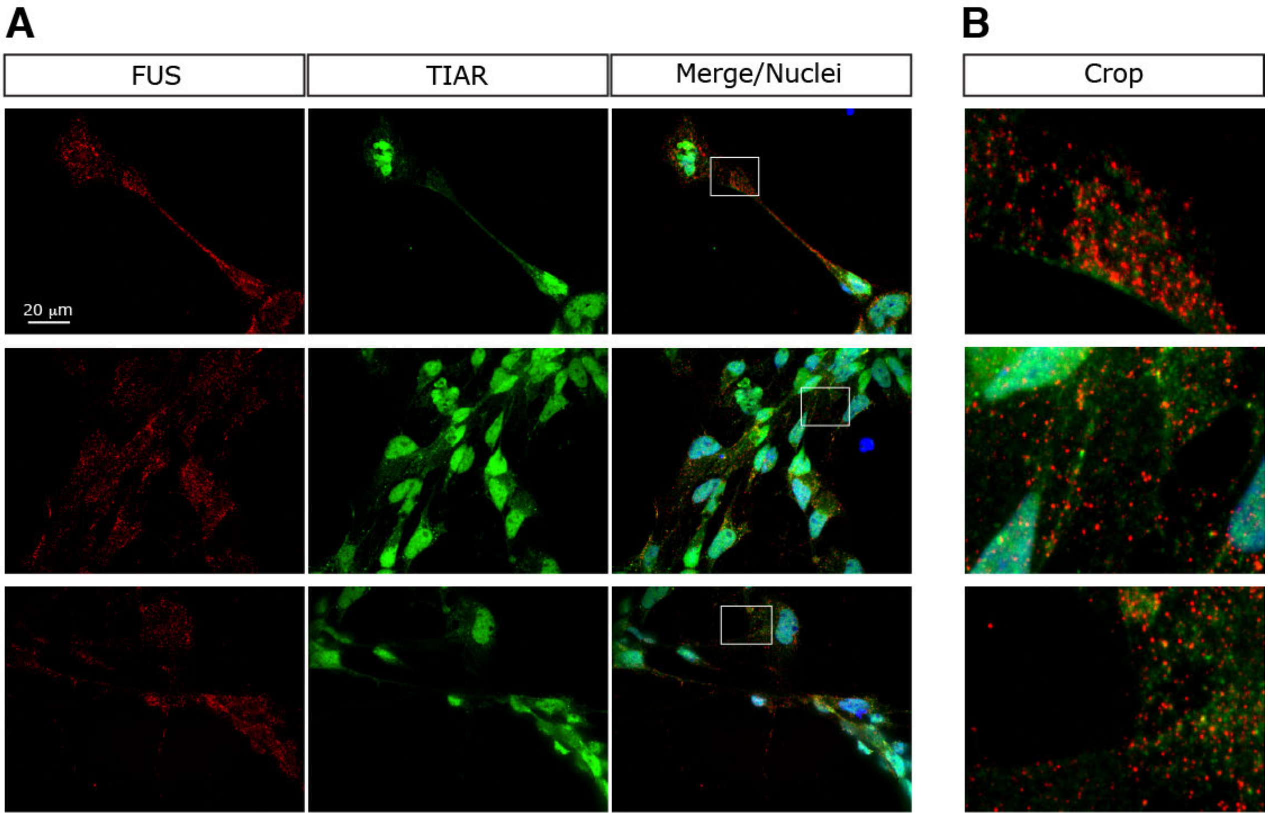

Figure S12 – FUS^{P525L} localization in differentiated iPSCs

(**A**) Immunostaining of FUS (red) and the stress granule marker TIAR (green) in iPSC FUS$^{P525L/P525L}$ differentiated for 34 days. Three representative images are shown. (**B**) Enlargements of the areas indicated with a white box in the Merge/Nuclei panels. Note that in the absence of stress cytoplasmic mutant FUS do not co-localize with TIAR. Blue: DAPI. Scale bar for all panels in (A) is 20µm.

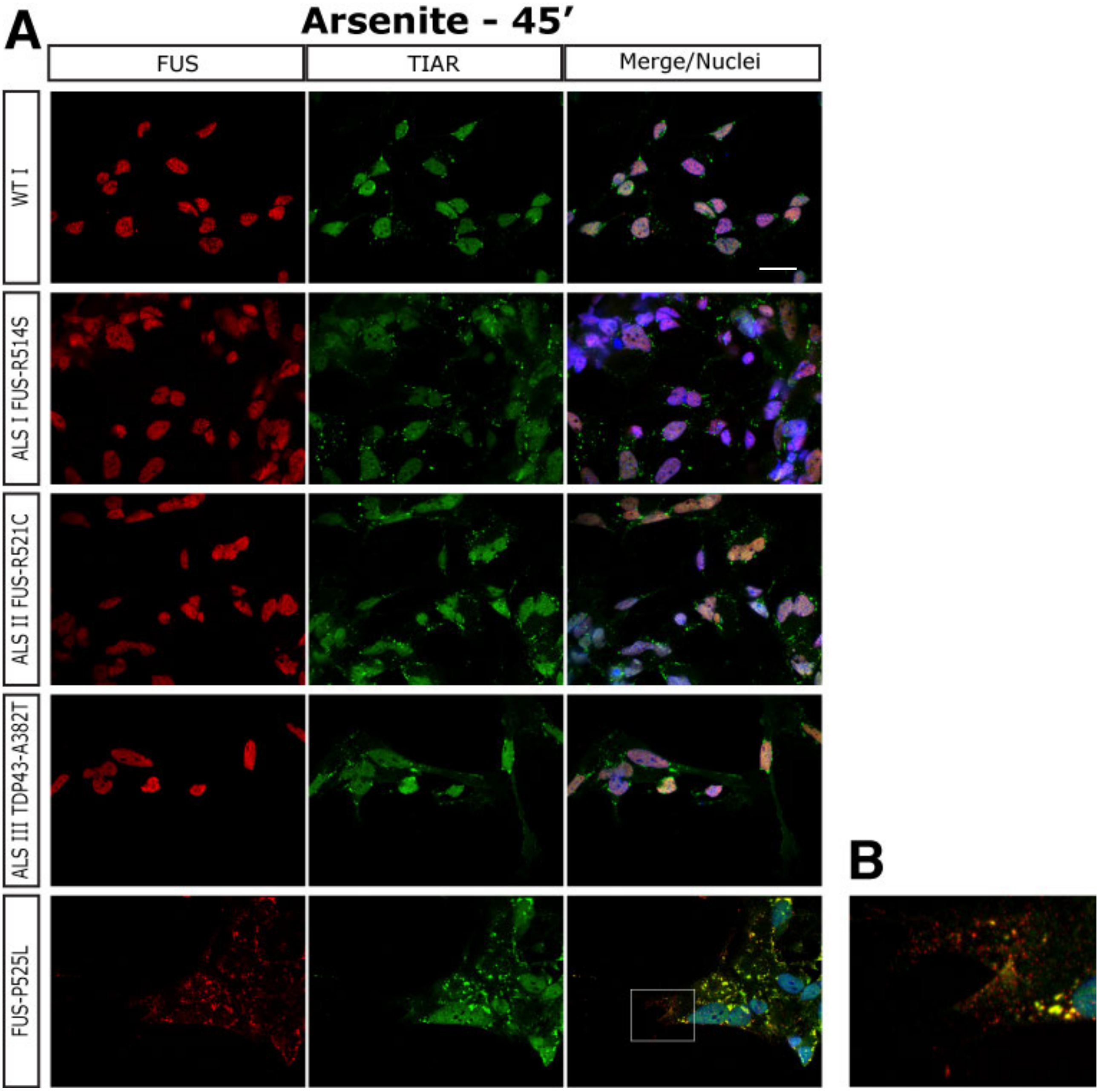

Figure S13 – FUS localization in differentiated iPSCs after 45 minutes of oxidative stress
(**A**) Immunostaining of FUS (red) and the stress granule marker TIAR (green) in WT and mutant FUS iPSCs differentiated for 34 days and treated with 0.5mM sodium arsenite for 45 minutes. FUS-P525L indicates the homozygous mutant. Representative images are shown. Blue: DAPI. Scale bar for all panels in (A) is 20µm. (**B**) Enlargement of the area indicated with the white box.

Note that at this earlier time point of arsenite treatment TIAR-positive SGs were already formed. FUS signal in these granules was barely detectable in $FUS^{R514S/WT}$ and $FUS^{R521C/WT}$ cells. In $FUS^{P525L/P525L}$ cells, even if FUS clearly localized in some SGs, a fraction of the protein

had not yet been incorporated. This evidence suggests that cytoplasmic mutant FUS recruitment into SGs is a late event during the formation of these structures.

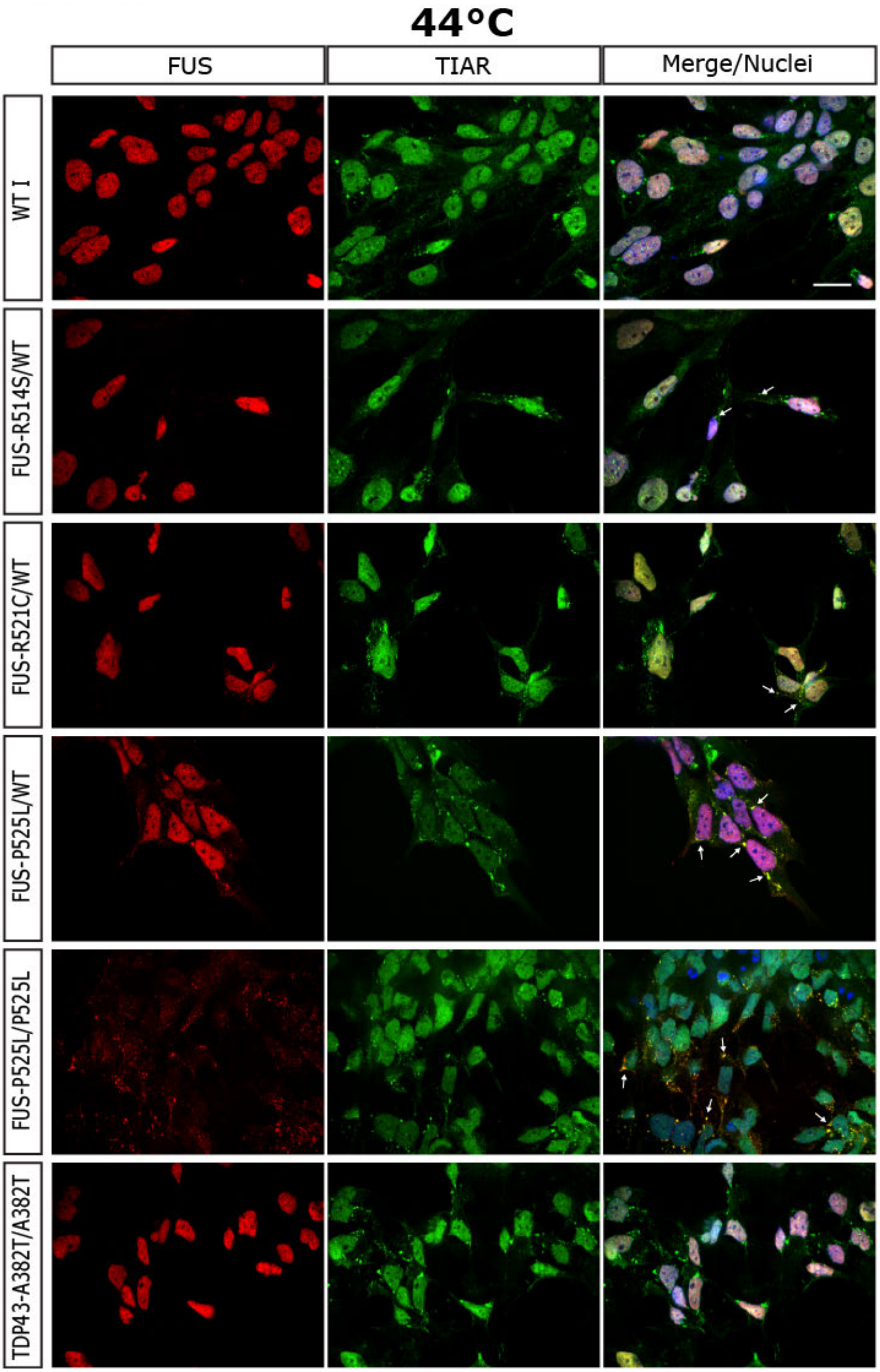
44°C
FUS
TIAR
Merge/Nuclei
WT I
FUS-R514S/WT
FUS-R521C/WT
FUS-P525L/WT
FUS-P525L/P525L
TDP43-A382T/A382T

Figure S14 – Mutant FUS localized in SGs during heat shock in differentiated iPSCs

Immunostaining of FUS (red) and the stress granule marker TIAR (green) in iPSCs differentiated for 34 days and cultured at 44°C for one hour. Blue: DAPI. Scale bar for all panels is 20µm.

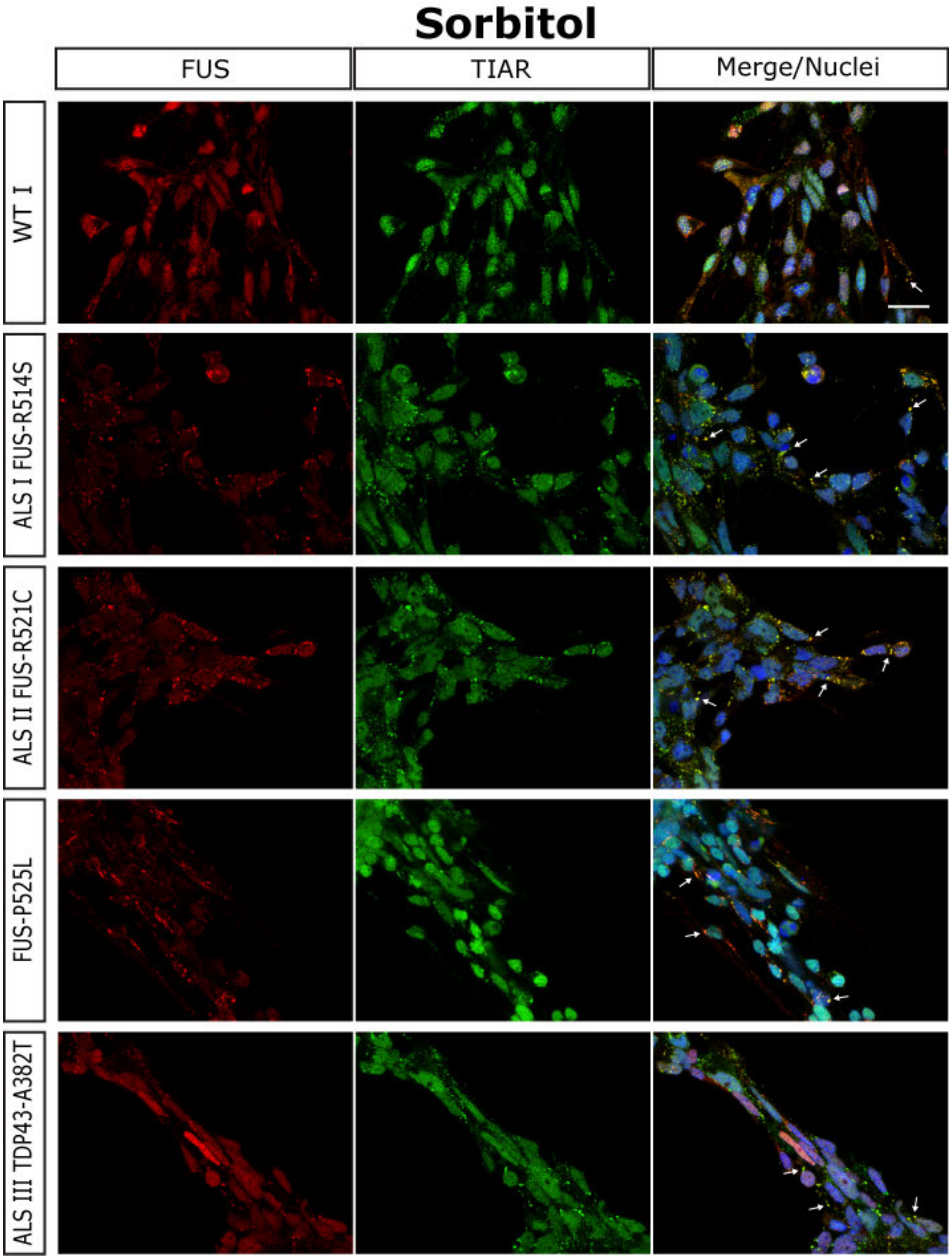

Figure S15 – Mutant FUS localization during hyperosmolar stress in differentiated iPSCs
Immunostaining of FUS (red) and the stress granule marker TIAR (green) in iPSCs differentiated for 34 days and cultured in presence of 0.4M sorbitol for 90 minutes. Blue: DAPI. Scale bar for all panels is 20µm. Upon treatment with sorbitol, WT FUS was detected in neural cells SGs to a minor extent than the mutants. Note, in sorbitol-treated cells, the reduced nuclear FUS signal in ALS I, ALS II and FUSP525L (homozygous mutant) compared to WT and ALS III cells.

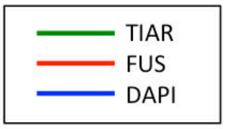

WT I

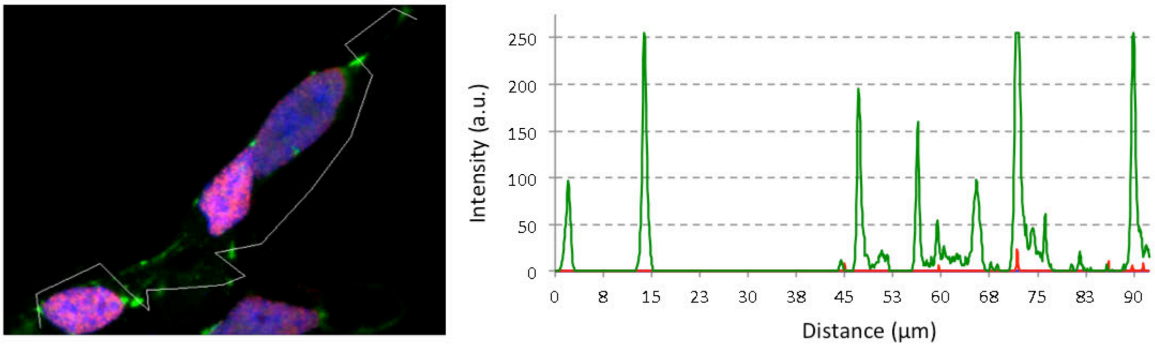

**FUS-
R514S/WT**

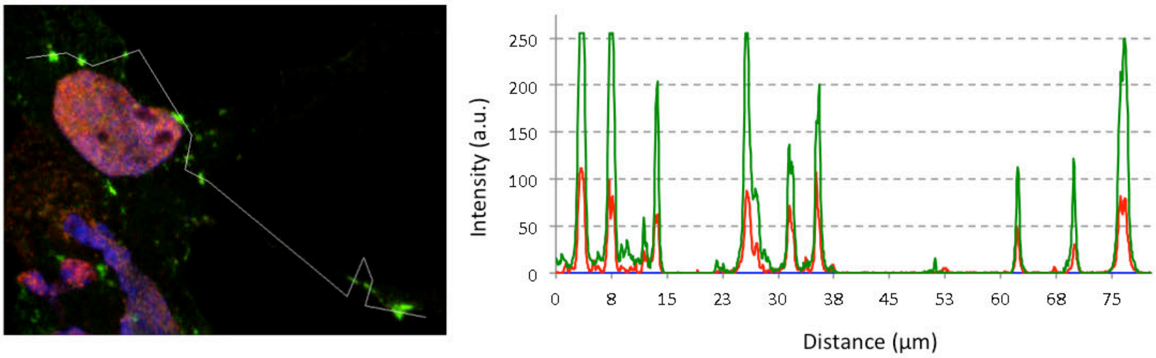

**FUS-
R521C/WT**

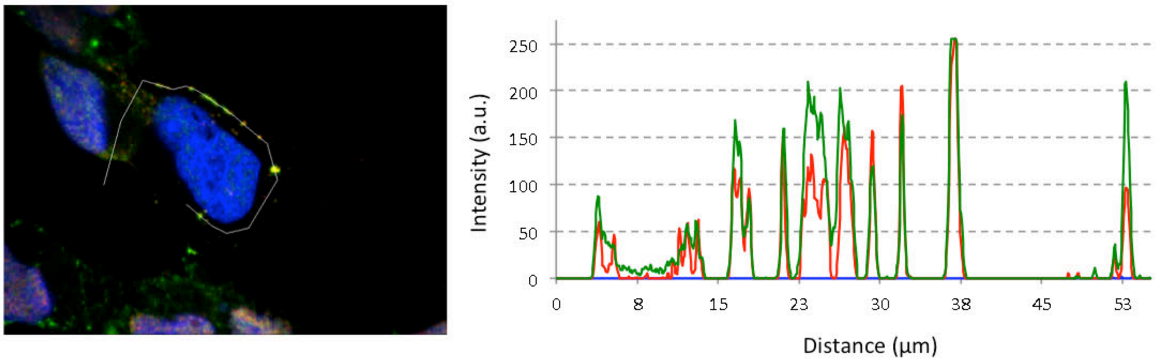

**FUS-
P525L/WT**

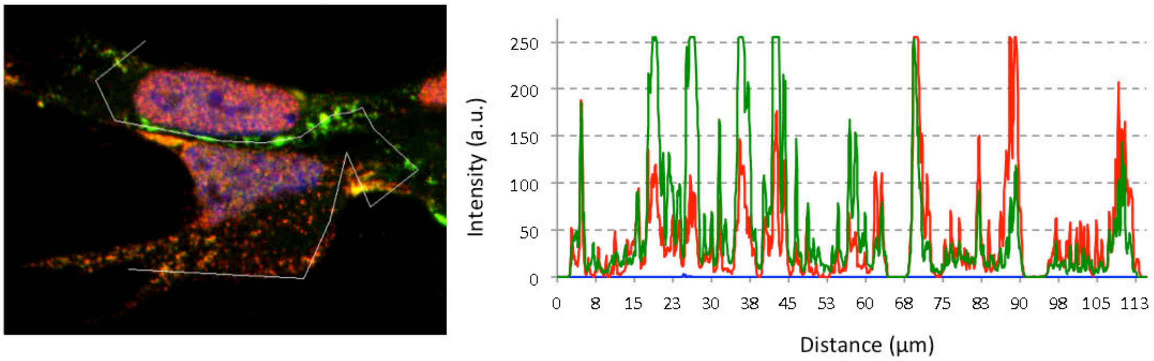

**FUS-
P525L/P525L**

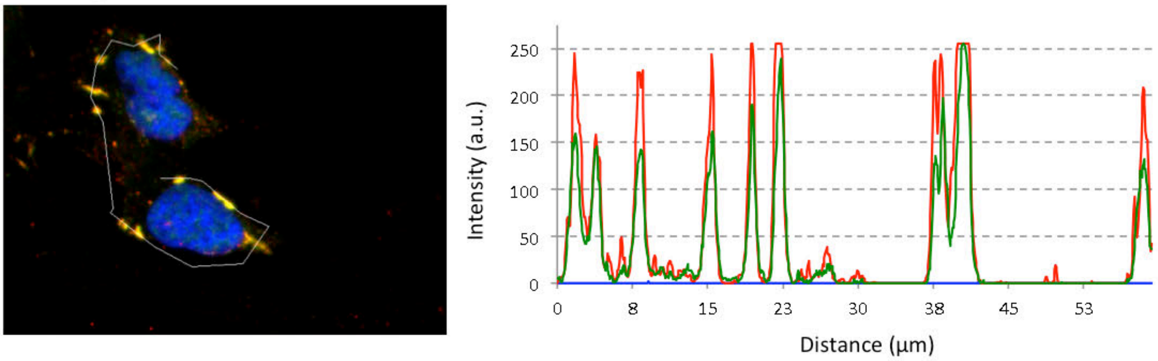

**TDP43-
A382T/A382T**

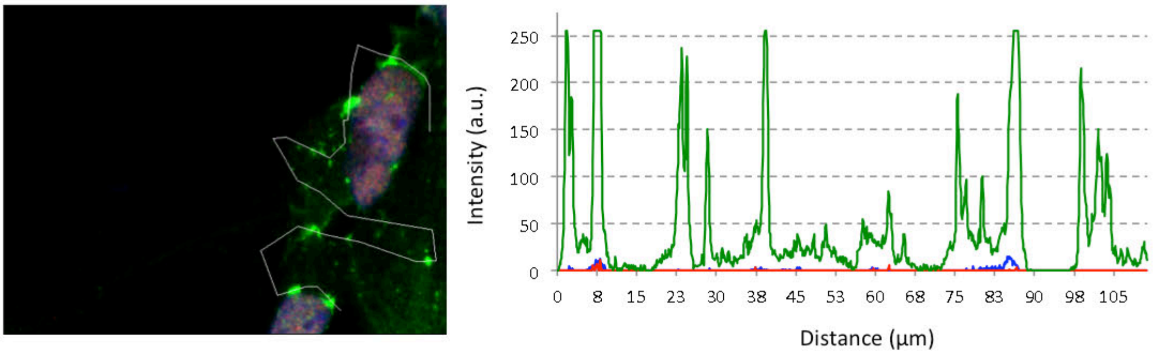

Figure S16 – Linescan analysis of ARS-treated differentiated iPSCs

Co-localization of FUS and TIAR within SGs was analyzed by linescan (see details in the Methods section). In each IF panel shown on the left, a line crossing TIAR-positive SGs was drawn. On the right, the graphs show the corresponding signal intensities of FUS (red), TIAR (green) and DAPI (blue), in arbitrary units, along the line.

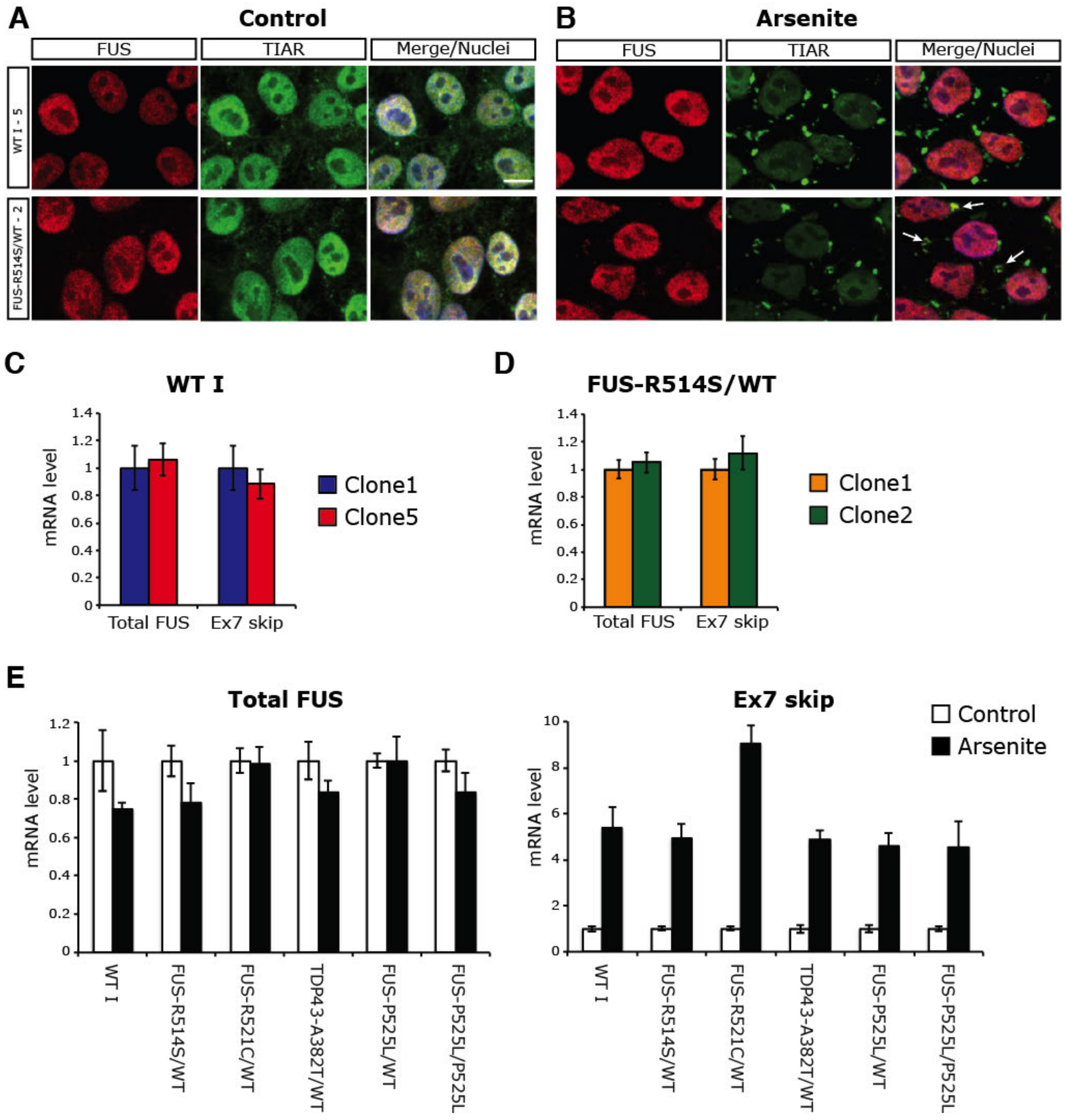

Figure S17 – Analysis of clonal variability in iPSC lines and analysis of FUS levels upon oxidative stress.

(**A-D**) Clonal variability was analyzed by comparing clone#1 and clone#5 of the WT I iPSC line and clone#1 and clone#2 of the ALSI-FUS$^{R514S/WT}$ iPSC line. Immunostaining of FUS (red) and TIAR (green) in undifferentiated iPSCs in control (untreated) conditions (**A**) or upon 0.5mM arsenite-induced oxidative stress for 60 minutes (**B**) showed no differences with clones shown in Figure 4. (**C-D**) FUS mRNA levels (total and devoid of exon 7), assessed by real-time qRT-PCR, were comparable in different clones. (**E**) Real-time qRT-PCR analysis of

FUS total mRNA (left) and alternatively spliced mRNA devoid of exon 7 (right) in iPSC lines in untreated conditions (Control) or after 60 minutes exposure to 0.5mM arsenite (Arsenite). Values are relative to Control samples for each iPSC line. The clearest effect of oxidative stress was an increase of the alternatively spliced form of FUS devoid of exon 7 in all lines. This variant contains a premature termination codon and is subject to Nonsense Mediated Decay (NMD) (Zhou et al., PLOS Genetics 2013), which depends from protein translation. As translation is blocked during stress, the simplest interpretation of the result is that the observed increase is indirectly induced by a temporary impairment of NMD in these conditions.

Supplementary methods - analysis of the off-target effects of the FUS C-term TALENs

In order to analyze possible off-targets effects of the TALEN pair used in this study, we have taken advantage of the PROGNOS (Predicted Report Of Genome-wide Nuclease Off-target Sites) tool, designed to aid in the prediction and analysis of the off-target effects of engineered nucleases (Fine et al., NAR 2013). The hg19 assembly of the human genome was used as reference. The parameters were as follows: up to 6 mismatches were allowed in each nuclease half-site and a spacing distance ranging from 10 to 30 nucleotides. Both homodimers and heterodimers of the nucleases were included in the analysis. A summary of the PROGNOS prediction is shown below. PROGNOS reported only one site in the human genome devoid of any mismatch, which corresponded to the FUS target site. This site ranked as the top 1 in a list of 2772 total sites. The vast majority of the possible off-targets contained 6 and 6 mismatches (1975/2772, about 71%) or 5 and 6 mismatches (619, about 22%). A very small fraction of potential off-targets, about 2%, maps to exons (40) or promoters (16).

PROGNOS Summary for 09092014SZB67xug_QUEUE_Ranking-TALENv2.0

```
The hg19 genome was searched for the TALEN pair targeting:
5'- TCAAATATAATGGATACTTA NN..NN TTTGCAGGGGTGAGCACAGA -3'

Using these RVDs:
Left:   01HD02NI03NI04NI05NG06NI07NG08NI09NI10NG11NN12NN13NI14NG15NI16HD17NG18NG19NI
Right:  01HD02NG03NN04NG05NN06HD07NG08HD09NI10HD11HD12HD13HD14NG15NN16HD17NI18NI19NI

Up to 6 mismatches were allowed in each nuclease half-site.

Allowed spacing distances were: 10 11 12 13 14 15 16 17 18 19 20 21 22 23 24 25 26 27 28 29 30

Homodimers and heterodimers of the nucleases were included in the output
```

2772 total sites were located	
**Mismatch Types**	**Genomic Regions**
0 and 0: 1	
3 and 4: 1	
3 and 5: 1	
4 and 4: 2	Exons: 41
3 and 6: 6	Promoters: 16
4 and 5: 18	Introns: 1024
4 and 6: 69	Intergenic: 1691
5 and 5: 80	
5 and 6: 619	
6 and 6: 1975	

We aimed at experimentally validating the absence of modifications, due to TALEN off-targeting, in the top 10 ranked sites, in the heterozygous FUS-P525L/WT and the homozygous FUS-P525L/P525L clones used in this study. The corresponding genomic sequences were PCR amplified and sequenced. We were able to amplify 8 sites from both iPSC lines. The remaining two sites are located in repetitive regions and could not be amplified from the parental WT I

line as well. TALEN off-targeting might result in small insertions or deletions originating from non-homologous end joining (NHEJ) of the cleaved DNA. Sequencing results showed absence of deletions or insertions in either clone for all sites analyzed (data not shown). Occasionally, we found single nucleotide differences, in both clones, compared to the reference genome. In all cases these corresponded to known polymorphisms. The alignment of the sequences for the rank 2 site is shown below as an example.

```
CLUSTAL 2.1 multiple sequence alignment


Genome              TATAAGTCTAAATGGATGAATGCGTGGAGTGAACCCTTCCATTTGTTTTAAGAATTGATC 60
FUS-P525L/P525L     ------------------------------------------------------------
FUS-P525L/WT        ------------------------------------------------------------

Genome              CATAAAGCACAACTAACTAGCTCATTTTTTTTCTGACTTGTCTGCCGGGAAAGTGCTGAT 120
FUS-P525L/P525L     ---------------ACTAGCTCATTTTTTTTCTGACTTGTCTGCCGGGAAAGTGCTGAT 45
FUS-P525L/WT        ---------------ACTAGCTCATTTTTTTTCTGACTTGTCTGCCGGGAAAGTGCTGAT 45
                                   *********************************************

Genome              CTGAGTCTGCTCTTCTTATTTTCCTTAACCCATAAAAAATGTCAAATGAAATGGATACAT 180
FUS-P525L/P525L     CTGAGTCTGCTCTTCTTATTTTCCTTAACCCATAAAAAATGTCAAATGAAATGGATACAT 105
FUS-P525L/WT        CTGAGTCTGCTCTTCTTATTTTCCTTAACCCATAAAAAATGTCAAATGAAATGGATACAT 105
                    ************************************************************

Genome              AGCTATGATTTCATTTCCATTATGCATTCATTACAGTTGAGATAAGGTGGAAACTTGGGG 240
FUS-P525L/P525L     AGCTATGATTTCATTTCCATTATGCATTCATTACAGTTGAGATAAGGTGGAAACTTGGGG 165
FUS-P525L/WT        AGCTATGATTTCATTTCCATTATGCATTCATTACAGTTGAGATAAGGTGGAAACTTGGGG 165
                    ************************************************************

Genome              CTTATTTATTCTAGAGTCTGTCGCAGATCATATCTCACCCTCTGCTCCCCACCTCATAGT 300
FUS-P525L/P525L     CTTATTTATTCTAGAGTCTGTCGCAGATCATATCTCACCCTCTGCTCCCCACCTCATAGT 225
FUS-P525L/WT        CTTATTTATTCTAGAGTCTGTCGCAGATCATATCTCACCCTCTGCTCCCCACCTCATAGT 225
                    ************************************************************

Genome              AATCTCAGTGCAGCTCTGTGTAGTACATGTCAGTGGGCTGTGTACTCCAATGACCCAGAG 360
FUS-P525L/P525L     AATCTCAGTGCAGCTCTGTGTAGTACATGTCA---------------------------- 257
FUS-P525L/WT        AATCTCAGTGCAGCTCTGTGTAGTACATGTCAG--------------------------- 258
                    ********************************

Genome              GACATCACATACATAGATGTCATTGTGGATAGTTTGATAAAGATTTCTAAATGGCAAA 418
FUS-P525L/P525L     ----------------------------------------------------------
FUS-P525L/WT        ----------------------------------------------------------
```

SUPPLEMENTARY TABLE 1

Oligos for real-time qRT-PCR

Gene	Oligo Forward	Oligo Reverse
REX1	AAAGCATCTCCTCATTCATGGT	TGGGCTTTCAGGTTATTTGACT
NANOG	CCAAATTCTCCTGCCAGTGAC	CACGTGGTTTCCAAACAAGAAA
SOX2	TCAGGAGTTGTCAAGGCAGAGAAG	GCCGCCGCCGATGATTGTTATTAT
DNMT3B	AATGTGAATCCAGCCAGGAAAGGC	ACTGGATTACACTCCAGGAACCGT
OCT4 end	ATGCATTCAAACTGAGGTGCCTGC	AACTTCACCTTCCCTCCAACCAGT
OCT4 hST	TCTGGGCTCTCCCATGCATTCAAA	CTGACAGCCATTGGACCTGGATTT
ATP5O	ACTCGGGTTTGACCTACAGC	GGTACTGAAGCATCGCACCT
HB9	GAGACCCAGGTGAAGATTTG	CCTTCTGTTTCTCCGCTTCC
OLIG2	GACAAGCTAGGAGGCAGTGG	CGGCTCTGTCATTTGCTTCT
ISL-1	TACAAAGTTACCAGCCACC	GGAAGTTGAGAGGACATTGA
CHAT	TCATTAATTTCCGCCGTCTC	GAGTCCCGGTTGGTGGAGT
FUS Exon 6-8	CAGAGTGGTGGAGGTGGCAGCG	CTTGGTCCCGAGGGCCCCATG
FUS (Total)	TCAGCTAAAGCAGCTATTGACTGG	GCCACCACCCCGATTAAAGTCTGC
FUS Ex15-3'UTR*	GGTGGTGGGGACAGAGGTGG	AATAACGAGGGTAACACTGGG
Chr4*	TTATCTTGTGGATGTTAG	AATCATGCAGATAATGAC

*for genomic DNA

SUPPLEMENTARY TABLE 2

Oligos for RT-PCR

Gene	Oligo Forward	Oligo Reverse
NR2F2	GCCATAGTCCTGTTCACCTCA	AATCTCGTCGGCTGGTTG
NESTIN	GCGTTGGAACAGAGGTTGGA	TGGGAGCAAAGATCCAAGAC
NCAM	ATGGAAACTCTATTAAAGTGAACCTG	TAGACCTCATACTCAGCATTCCAGT
BRACHYURY	CACCTGCAAATCCTCATCCTCAG	TGTCATGGGATTGCAGCATGGA
RUNX1	CCCTAGGGGATGTTCCAGAT	TGAAGCTTTTCCCTCTTCCA
MIXL1	GGTACCCCGACATCCACTT	GCCTGTTCTGGAACCATACCT
GATA4	CTAGACCGTGGGTTTTGCAT	TGGGTTAAGTGCCCCTGTAG
AFP	AGCTTGGTGGTGGATGAAAC	CCCTCTTCAGCAAAGCAGAC
SOX17	GGCGCAGCAGAATCCAGA	CCACGACTTGCCCAGCAT
GAPDH	CACCATCTTCCAGGAGCGAG	CCTTCTCCATGGTGGTGAAGAC

SUPPLEMENTARY TABLE 3

Oligos for cloning FUS HDR donor vectors

Gene	Oligo Forward	Oligo Reverse
FUS gDNA DONOR	AAAGGCAGACCTGGTGCTAG	TCCACAAACTCCTGCAGCACT
FUS P525L DONOR	TTAGCCTGGCTCCCCAGGTTC	TACaGCCTCTCCCTGCGATCC
FUS WT DONOR	TTAGCCTGGCTCCCCAGGTTC	TACGGCCTCTCCCTGCGATCC
eGFP-PURO DONOR	TGTGAGCAAGGGCGAGGAGCT	TCAGGCACCGGGCTTGCGGG
PB Pu delta Tk DONOR	TTAACCCTAGAAAGATAATCA	TTAACCCTAGAAAGATAGTCT
